# Assessing the Environmental Impacts of Microfluidic Devices for Glucose Detection

**DOI:** 10.1021/acssuschemeng.5c01511

**Published:** 2025-06-18

**Authors:** Kristie J. Tjokro, Valerio Barbarossa, Stefano Cucurachi, Alina Rwei, Justin Lian

**Affiliations:** † Institute of Environmental Sciences (CML), 4496 Leiden University, Einsteinweg 2, Leiden 2333 CC, The Netherlands; ‡ Global Sustainability, PBL Netherlands Environmental Assessment Agency, Bezuidenhoutseweg 30, The Hague 2500 GH, The Netherlands; § Department of Chemical Engineering, 2860 Delft University of Technology, Van der Maasweg 9, Delft 2629 HZ, The Netherlands

**Keywords:** ex ante, emerging technologies, rapid prototyping, process scale-up, lab-on-a-chip, polydimethylsiloxane, 3D printing, polylactic acid

## Abstract

Healthcare must balance safety, efficiency, and effectiveness with affordability and accessibility. Microfluidic devices offer low-cost, portable solutions for point-of-care testing, miniaturizing lab functions on chips through microchannels for quick diagnostics, retaining resolution and sensitivity with minimal reagent use. However, their environmental sustainability is uncertain, with concerns about production scale-up, risks from disposability, and the impact of alternative raw materials or manufacturing techniques compared to traditional soft lithography based on polydimethylsiloxane (PDMS). We conducted a cradle-to-grave life-cycle assessment (LCA) of three glucose-detection devices, a PDMS device via soft lithography, a paper device via wax stamping, and a polylactic acid (PLA) device via 3D printing, for both laboratory-scale and commercial-scale production. For lab-scale production, the paper device had the lowest environmental impact across most impact categories, while the PLA device had the highest. However, for commercial-scale production, by transitioning from 3D printing to injection molding, the PLA device performed best overall, while PDMS performed the worst. For both scales, material and energy use were key contributors, with minimal impact from the use phase. This study highlights the importance of considering environmental impacts at multiple scales and shows the added value of using LCA to guide design and production for early-stage technologies.

## Graphical abstract

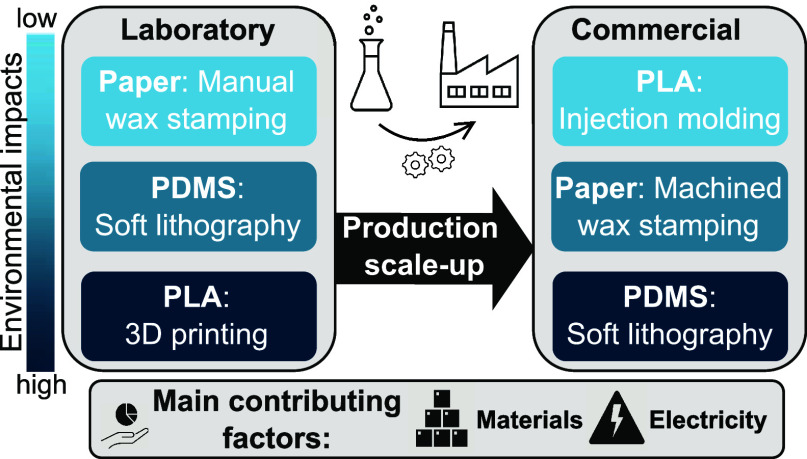

## Introduction

A challenge surrounding healthcare is to ensure that it is safe, efficient, and effective, while minimizing costs and maintaining accessibility.^1^ Microfluidic devices for point-of-care applications are a potential low-cost method to increase the accessibility of healthcare in areas with low access to medical laboratories.^2^ With microfluidics, laboratory functions such as detection and separation can be condensed onto a miniature device consisting of various microchannels, through which tests like diagnostics can be performed.^3^ Furthermore, as smaller volumes of reagents and samples are required, these tests are more portable, cheaper, and quicker to perform, while retaining high resolution and sensitivity.^3^

Yet, there are many unknowns regarding microfluidic devices in terms of their environmental sustainability. Mainly, it is unclear whether these devices are commercially viable while remaining environmentally sustainable.^4^ Microfluidic devices for point-of-care applications are mainly produced in small-scale proof-of-concept studies using soft lithography on polydimethylsiloxane (PDMS), an optically transparent, soft elastomer.^5^ The production of PDMS devices on a laboratory scale is resource and energy-intensive: soft lithography requires silicon molds, which are manufactured using specialized equipment, often requiring energy-intensive cleanrooms.^6^ Additionally, while soft lithography in PDMS is suitable for rapid prototyping, it does not scale well for commercialization due to its relatively high cost and complex manufacturability.^7^ Another unknown is the potential risk that microfluidic devices could pose to the environment in their end-of-life since they are usually designed to be disposable and single-use. For example, PDMS is difficult to recycle once cured, cannot be remolded into a new part, and is not biodegradable.^8^

Research on improving the sustainability of microfluidic devices has resulted in studies focusing on alternate materials to PDMS with lower environmental footprints, such as poly­(methyl methacrylate) (PMMA),^9,10^ polylactic acid (PLA),^11,12^ and corn proteins.^13^ Other alternative materials include paper;^14–16^ plastics, like acrylics, polystyrene, and polytetrafluoroethylene (PTFE); hydrogels,^17^ and textiles.^18^ Alongside these materials, alternative manufacturing methods have emerged, like the 3D printing of (bio)­plastics^19^ and wax printing on paper.^20^ These alternatives often offer flexibility and cost savings over soft lithography on PDMS.^21^ This highlights the multifaceted nature of microfluidic devices’ environmental sustainability.

Here, we use ex-ante life cycle assessment (LCA)^22^ to assess how choices in material and manufacturing methods influence the environmental impacts associated with microfluidic devices for point-of-care applications of glucose testing for humans. We chose this application given the importance of glucose monitoring with diabetes increasingly becoming a global issue.^23^ We consider three microfluidic devices: (1) a PDMS device made using soft lithography, (2) a paper device made using wax stamping, and (3) a PLA device made using 3D printing. These devices represent traditional, emerging, and biodegradable materials, respectively, in the microfluidics field. For each device, we conduct an LCA for production scenarios at both laboratory-scale and commercial-scale to understand whether environmental impacts change when scaling up the manufacturing process. This study highlights the importance of performing an LCA on technologies at the proof-of-concept stage to guide future design decisions. Our results provide useful insights for health practitioners and researchers that can help understand how microfluidics devices can be designed more sustainably.

## Materials and Methods

### Devices Selection

We assessed the environmental performance of three microfluidic devices developed for colorimetric glucose detection using comparative LCA. The materials we assessed were PDMS based on the work of Koh et al.,^24^ paper based on the work of Gabriel et al.,^25^ and PLA based on the work of Tothill,^26^ using soft lithography, wax stamping, and 3D printing, respectively. These devices have similar sizes, use similar colorimetric reagents to detect glucose (see the details of the glucose detection principles for each device in the Supporting Information SI-S1), and are applicable to similar samples. They were chosen based on the available information on their compositions and manufacturing methods, their similarities, and their complexity relative to other glucose-detecting microfluidic devices. Considering the studies on the three devices were all published between 2016 and 2017, they are contemporaries and represent the state-of-the-art in their field. This further facilitates comparability between devices. All information on manufacturing and use can be found in the papers of the respective authors.

As these devices are not commercially produced, we based their designs on recent proof-of-concept papers. We chose accessible, low-complexity production methods suitable for lab-scale manufacturing. Device specifications, i.e., manufacturing information, and detailed product system flowcharts, can be found in SI-S1.

### PDMS Device

According to Koh et al.^24^ (Figure 1A), the PDMS device is disk-shaped, has a diameter of 3 cm, a thickness of 700 μm, and consists of three layers of PDMS. The middle layer contains the microfluidic channels carrying the reagents and colorimetric dyes. The device is designed to be worn on the skin and has inlets through which the sweat of the user can enter the microchannels to cause a colorimetric reaction. The bottom layer serves as the skin-adhesive and the top layer encloses and protects the reagents.

**Figure 1:**
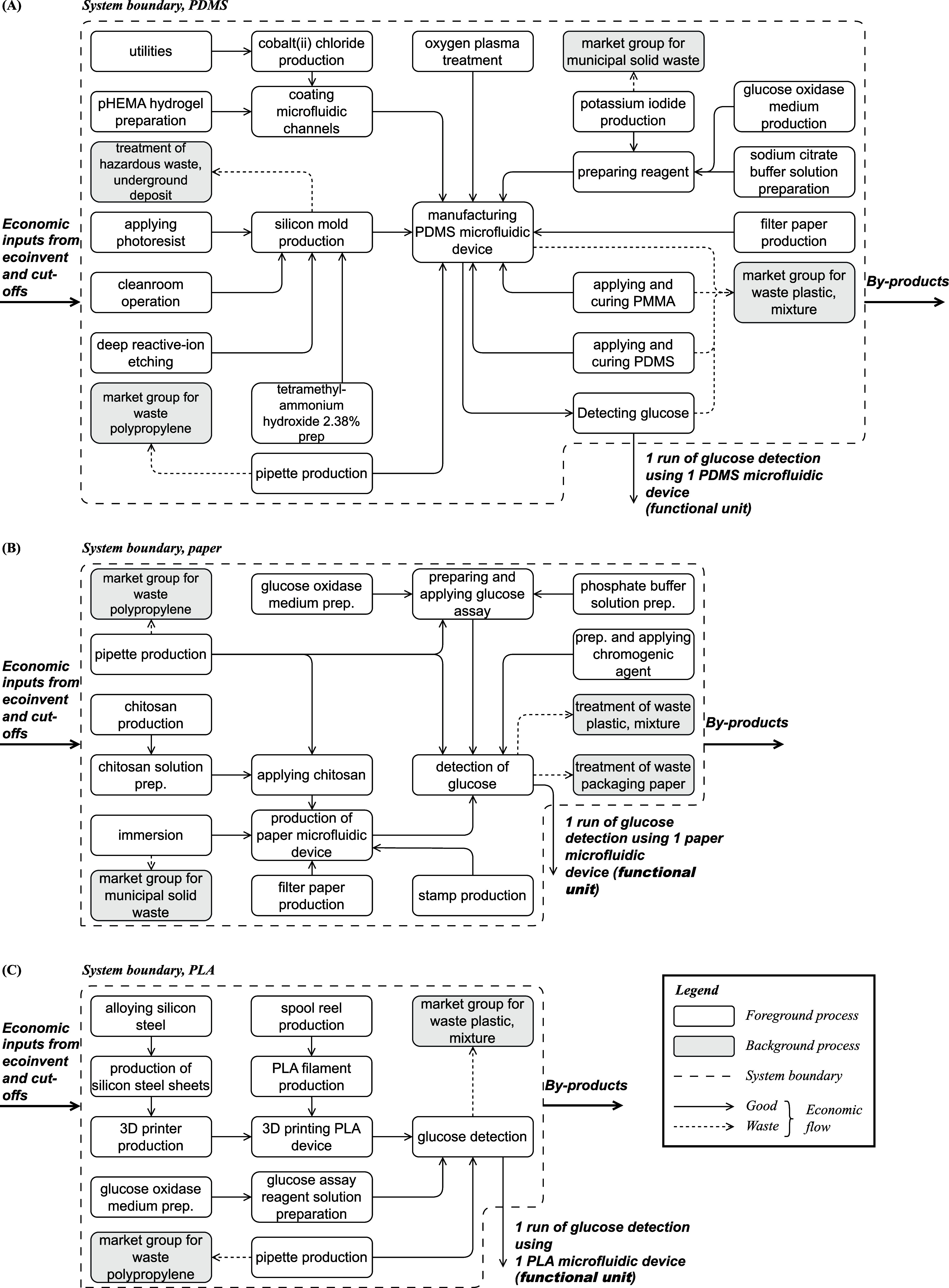
Product system flowcharts for (A) the PDMS device, (B) paper device, and (C) PLA device. The legend applies to all three product flowcharts. Detailed flowcharts can be found in SI-S2.2.

Koh et al.^24^ describe the device’s production process through replica molding. The mold is made in a cleanroom (25 m^2^, ISO5) on a silicon wafer, through soft lithography using a negative photoresist SPR 220 4.5, and deep reactive ion etching (DRIE) on a silicon wafer as a master.^24^ Data for the mold fabrication is based on expert estimates. In this work, we assumed the mold can be reused 10 times.^27^ First, PMMA is spin-cast onto the master (3000 rpm, 30 s) and cured at 180 °C for 5 min. Then, PDMS (30:1 ratio of base to curing agent by weight) is spin-cast onto the mold (200 rpm, 30 s) and is left to cure in an oven for 4 h at 70 °C. Then, the layers are bonded using oxygen plasma. The device is cooled until use, in order to preserve the enzymes within the device.

### Paper Device

Gabriel et al.^25^ (Figure 1B) describe the production method for a paper microfluidic device which colorimetrically estimates the glucose concentration in human tears. The authors incorporate chitosan – a sugar extracted from the outer skeletons of shellfish – to enhance color clarity on the paper which improves the analytical performance of the device.

The paper-based microfluidic device had a dimension of 45 mm by 45 mm and is based on two sheets of filter paper (grade 40, pore size 25 μm), one native sheet and one sheet impregnated with paraffin wax.^28^ The filter paper undergoes no pretreatment prior to impregnation with paraffin. They are joined through hand-held wax stamping using heat. This is a method described in the paper by de Tarso Garcia et al.,^28^ and it utilizes paraffin wax to form hydrophobic barriers in the filter paper to enclose the reactants. The chitosan and glucose assay are applied to the surface, allowing for drying at room temperature each time. We assumed that no specific storage conditions would be necessary for the paper device once dried.^28^

### PLA Device

The PLA device, based on Tothill^26^ (Figure 1C), is manufactured with PLA filament using a consumer-grade fused deposition modeling 3D printer. The device is disk-shaped with a diameter of 90 mm and a thickness of 2 mm. The device is printed at a speed of 10 mm s^–1^, with a layer height of 0.06 mm. The total printing time is 8.5 h and about 14 g of PLA filament is required per device. After printing, the device is treated with heat to ensure channel watertightness.

### Life Cycle Assessment

We applied LCA^29^ to determine the cradle-to-grave environmental impacts associated with the three alternative microfluidic devices for glucose detection. This includes raw material inputs, biochemical reagents, the use phase, and waste disposal. We defined the functional unit as one test to detect glucose in a human biological sample.

We assumed that the fabrication processes, use, and waste disposal occurred in the European economic region. Transport was accounted for if processes occurred on other continents, while transport between manufacturing sites and use was excluded. Production emissions are also excluded. We adopted the Environmental Footprint for the life cycle impact assessment,^30^ and used the Activity Browser LCA software^31^ for calculations. All the background data for the LCA was sourced from the ecoinvent 3.10 database.^32^ We collected foreground data from literature and patents. The electricity consumption of laboratory equipment was sourced from retailer Web sites. We did one direct measurement to determine the electricity consumption of an electric convection oven. For the production of silicon molds for the PDMS device, we interviewed an expert on soft lithography, due to a lack of available data in literature and patents. During this interview, the expert estimated values for the type and quantity of materials used during the process, based on their experience in the field. For all devices, we estimated the missing life cycle inventory data for chemicals using a stoichiometry-based approach.^33^ This includes chemical inputs and estimated energy demand but excludes water and storage needs. Detailed stoichiometry process calculations for chemicals and reagents and life cycle inventory data can be found in SI-S2.2 and SI-S2.3. The contribution analysis was performed using the Sankey functionality of the Activity Browser, which visualizes the most significant contributing unit processes for each environmental impact category. A 3% cutoff threshold was applied to focus on the most impactful processes.

### Scaling Up Production

We conducted the LCA on both laboratory and commercial scales. Due to limited data on scaling from laboratory to commercial production, we used a simple upscaling procedure and, therefore, excluded uncertainty analysis.^22,34^ The adjusted life cycle inventories can be found in SI-S3.

To model commercial-scale production, the product systems of the alternatives were assessed for potential improvement points. These include reducing generated waste and energy consumed per device produced by increasing throughput, e.g., by using more efficient production methods or larger equipment that enables higher-volume manufacturing. We examined to what extent improvements in these areas could reduce environmental impacts in the large-scale production scenario. If no potential improvements could be identified, an alternative manufacturing method was implemented.^35^

For the PDMS device, we assumed that less energy would be required per device produced. Assuming industrial machinery, device production would become more efficient: larger ovens and larger refrigerating units mean more simultaneous treatment, resulting in a smaller energy requirement per device. This also reduces space requirements per device, reducing cleanroom energy consumption per device produced. However, there would also be a need for larger cleanrooms (100 m^2^, ISO5) to produce more silicon molds.

For the paper device, we assumed that the hand-held stamping method would be automated using specialized equipment in an assembly line. This increased efficiency leads to reduced waste generation. We modeled this as a reduction in generated paraffin waste during the immersion process. However, automation introduces new resource and energy intensive processes, which is reflected in the model.

For the PLA device, we found that 3D printing would not be a viable method of production on a larger scale,^36^ as it is improbable for a 3D printer to achieve high daily production volumes. Rather than eliminating inefficiencies, we identified a conventional technology to replace 3D printing with. We assumed that the PLA device’s design and dimensions made it suitable for production via injection molding. This assumption is based on injection molding being cited as a viable alternative to 3D printing for large-scale production following rapid prototyping.^36,37^

## Results

### Characterization Results

Our assessment revealed that across all categories except ozone depletion, the laboratory-scale PLA device is associated with the highest emissions (Figure 2). Conversely, the device with the overall best environmental performance is the commercially produced PLA microfluidic device via injection molding (Figure 2). Figures S9 and S10 show the separate characterization results in relative terms for the laboratory and commercial-scale product systems, respectively. Tables S15 and S16 show the absolute values.

**Figure 2:**
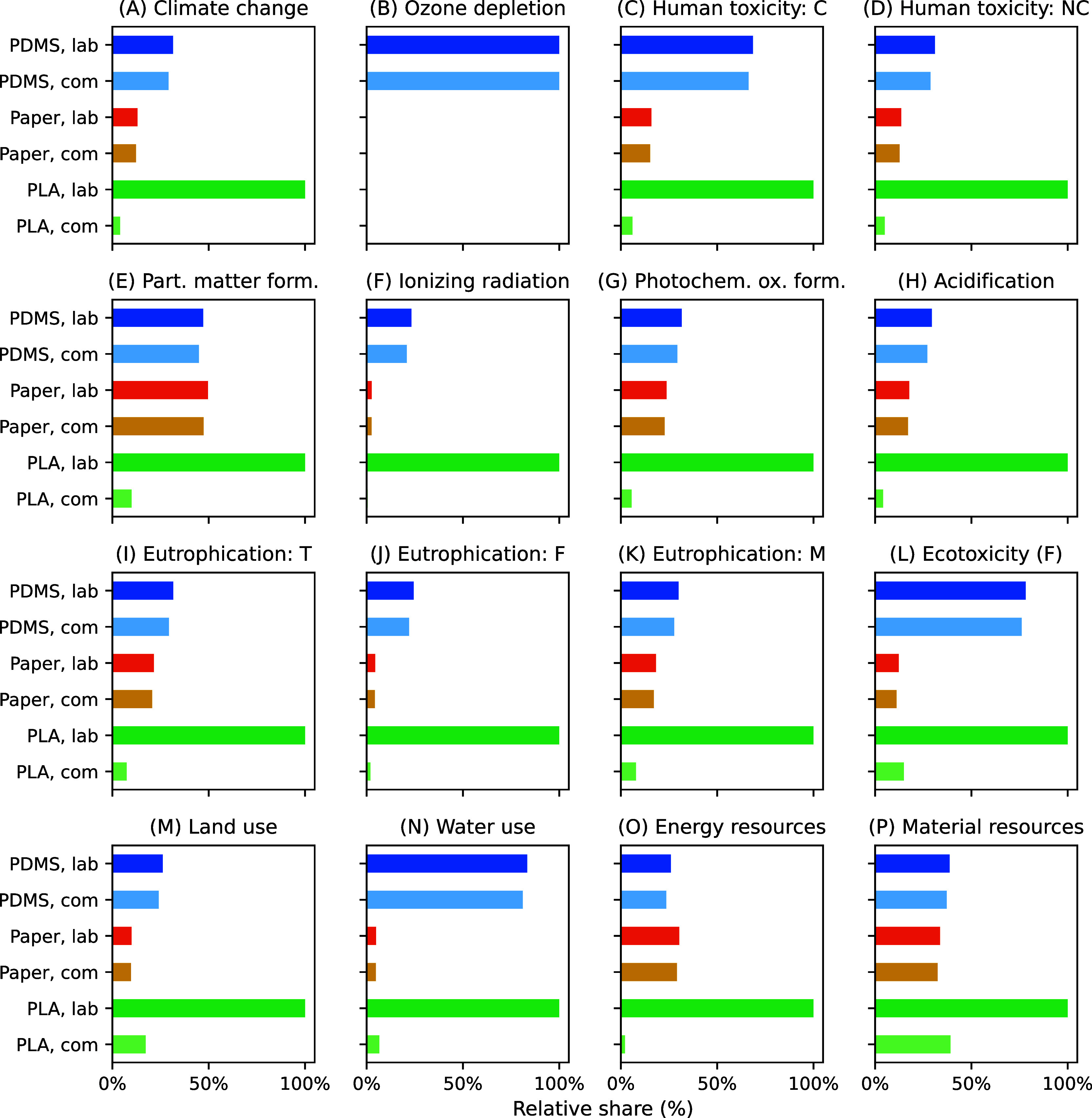
Characterization results for the three microfluidic devices PDMS, Paper and PLA, shown for both laboratory (lab) and commercial-scale (com) production. The impact magnitude is shown relative to the largest impact for each category: (A) climate change, (B) ozone depletion, (C) human toxicity: carcinogenic, (D) human toxicity: noncarcinogenic, (E) particulate matter formation, (F) ionizing radiation, (G) photochemical oxidant formation, (H) acidification, (I) eutrophication: terrestrial, (J) eutrophication: freshwater, (K) eutrophication: marine, (L) ecotoxicity: freshwater, (M) land use, (N) water use, (O) energy resources, (P) material resources.

The PDMS device ranks second in the laboratory scenario and third in the commercial scenario (Figures 2 and S10). Especially in the categories ozone depletion, ecotoxicity: freshwater, and water use, the difference between the PDMS device and the other alternatives is stark (Figure 2). The difference in environmental impacts between the two production scenarios is minimal. We found that the design of the device could only be retained in an upscaling scenario if the production method remained unchanged.

Assuming lab-scale production, the paper device shows the lowest environmental impact in all categories except particulate matter formation and energy resources, where the PDMS device performs slightly better (Figure 2). This is because paraffin’s production is heavily dependent on fossil fuels. The resulting emissions are slightly higher than those associated with the production of liquid oxygen and PDMS cumulatively (Figure 2). On a commercial scale, the paper device ranked second across most impact categories, except ecotoxicity: freshwater, land use, water use, and material resources, where it ranked first (Figure 2). Similarly to the PDMS device, upscaling production does not lead to drastic emission reductions.

On a lab scale, the PLA device performs significantly worse than the other devices across all impact categories (Figures 2 and S9). This is due to the high energy demands of 3D printing, a time-intensive process due to the layer-by-layer additive manufacturing process and the PLA design’s high precision requirements. However, there is a considerable difference in impacts between the lab and commercial production scenarios (Figures 2 and S9). This is because the product system for commercial production assumes the use of injection molding instead of 3D printing. While 3D printing had a high electricity demand, given the 8-h printing times, injection molding is significantly more efficient, explaining the drastic decrease in environmental impacts (Figures 2 and S9). Here, the PLA device performs best in all categories except ecotoxicity: freshwater, land use, water use, and material resources, where the commercial-scale paper device slightly outperforms the PLA device. This is because PLA is derived from maize grain, which has slightly higher emissions than the electricity and paraffin production required for the paper device.

The characterization results show that upscaling simplistic production processes can have minimal effects on the total emissions, resulting in low environmental advantages (Figure 2). This is because any potential reduction in environmental impacts is counterbalanced by the implementation of techniques that allow for automation and high throughput, which go paired with high emissions. This is particularly evident in the case of the paper device. The implementation of assembly line equipment contributes to the total emissions, whereas this was absent in the laboratory scenario. However, if production is increased, this effect is reduced. Additionally, waste generation is reduced on a larger scale, due to more efficient use of paraffin (Table S20). Unlike the PDMS and paper devices, upscaling production of the PLA device results in a substantial decrease in emissions per functional unit (Figure 2). This highlights that accounting for system changes in upscaling production is important as it can lead to considerable shifts in environmental impacts.

### Contribution Analysis

The two main factors contributing to the environmental impacts of the PDMS microfluidic device are the material polydimethylsiloxane itself and the oxygen required for the oxygen bonding plasma treatment (Figure 3). Electricity use and the silicon mold contribute to a lesser extent. This is true for both the laboratory and commercial production scenarios (Figure 3).

**Figure 3:**
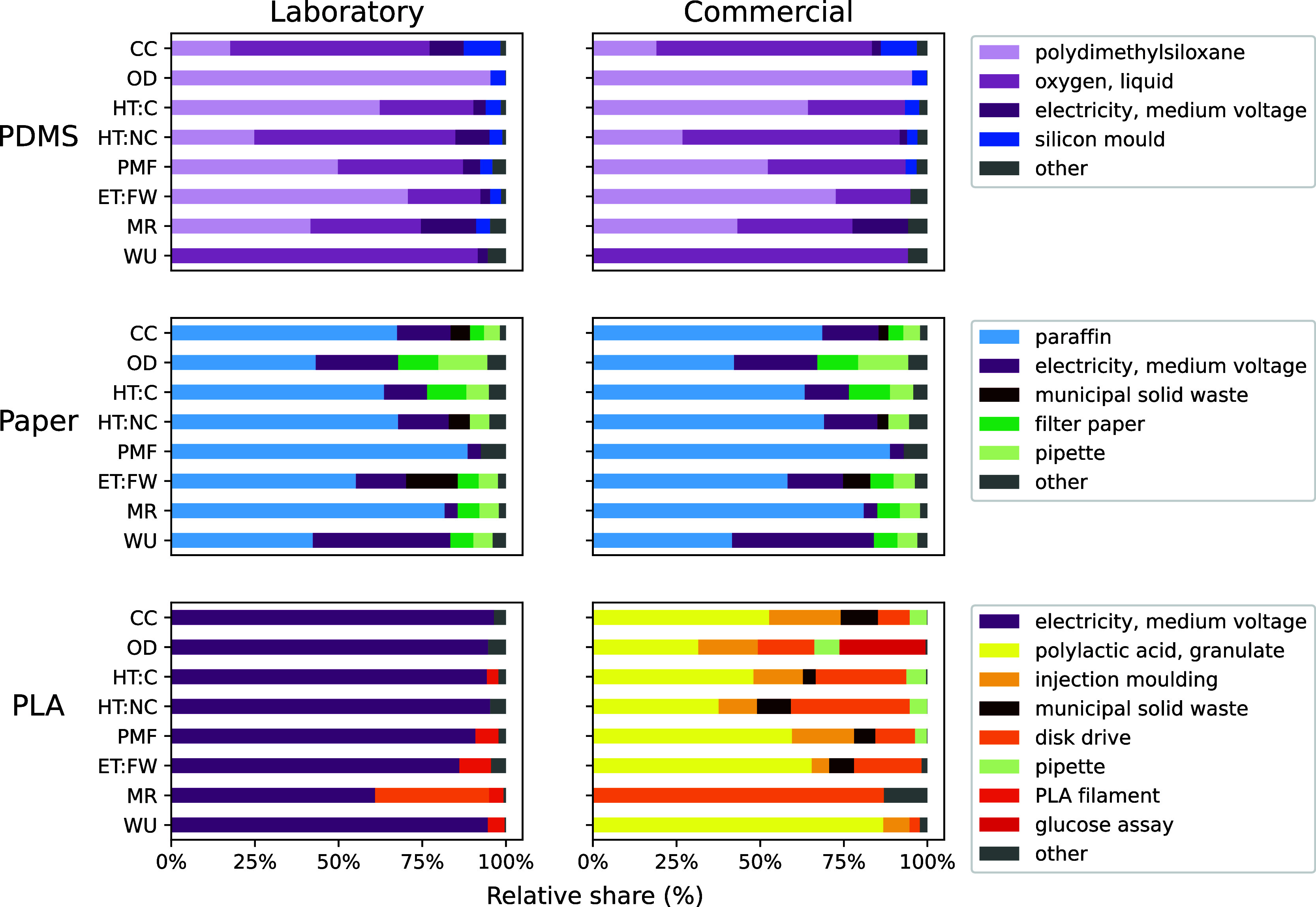
Contribution analysis for the three microfluidic devices, shown for both laboratory and commercial scale production across 11 selected impact categories. Contribution values in percentages are provided in SI-S4.2. The “other” category includes all elementary flows which do not make a contribution to the overall impacts larger than 3%. This might therefore include use-phase impacts. Impact categories are abbreviated as follows: CC: climate change, OD: ozone depletion, HT:C: human toxicity: carcinogenic, HT:NC: human toxicity: noncarcinogenic, PMF: particulate matter formation, ET:FW: ecotoxicity: freshwater, MR: material resources: metals/minerals, WU: water use.

The paraffin coating on the paper device, which creates a barrier to contain the sample and reagents, is the main contributor to the environmental impact categories, followed by electricity use and the paper material (Figure 3). The use phase contributes to the overall emissions through the pipettes used during analysis and the waste treatment of the discarded device. For the paper device, most gains in environmental performance can be achieved by transitioning to an electricity grid based on renewables rather than fossil fuels.

For the laboratory-scale PLA device, electricity used for 3D printing is the largest contributor (Figure 3). This is due to the long printing time, causing the emissions from electricity use to outweigh all other material requirements. For material resources, the disk drive makes the second most significant contribution in the laboratory-scale production scenario, and it is the most significant contributor in the commercial scenario. The PLA material itself is only responsible for a small part of the emissions, contrary to the other devices where the material inputs were more important factors (Figure 3). In contrast, at commercial-scale production, the PLA material itself is the largest contributor to impacts and electricity plays only a marginal role (Figure 3). The next most significant contributors are the use of a disk drive to conduct the analysis, especially in the material resources category, as well as the injection molding process (Figure 3). The glucose assay, which is necessary to perform the analysis, only makes a considerable contribution to the ozone depletion category (Figure 3). In the other product systems, chemical use contributes too little to be reflected in the contribution analysis.

## Discussion

### Device Performance

The characterization results and the contribution analysis showed that there are aspects of the impacts that can be tackled similarly across all three devices. First, electricity use is almost always a significant contributor (2.23–96.40% depending on device, scale, and impact category) to the overall environmental impact of the devices. This is in line with findings from other studies.^38–40^ By implementing renewable energy sources into the power supply, the impacts could be reduced for all three alternatives. The lab-scale PLA device would improve the most compared to the other devices, as electricity was the main contributor to its environmental impacts. For the other alternatives, impact reductions are minimal when switching to a renewables-based electricity supply.

Second, the three devices can achieve large reductions in emissions through redesigning (parts of) the device. This is especially true for the PDMS and paper devices. For the former, alternative recyclable materials that could improve the end-of-life of the device could be explored. These include thermoplastic elastomers^41^ and recyclable silicones.^42,43^ Another redesign opportunity is to avoid a master mold and potentially reduce generated PDMS waste by circumventing spin coating. This could be achieved by using an alternate manufacturing method, where the channels are formed into the PDMS directly instead of casted. Examples include laser cutting,^44^ xurography,^45^ and low-energy electron beam irradiation.^46^ Redesigning the device such that the reagents and enzymes are only applied before use, thereby removing the need for cooling, would also help reduce environmental impacts. Conversely, as the paper device has low complexity, there are fewer options to improve its environmental performance. One approach could be to explore alternative materials with lower environmental impact, like beeswax or plant-based alternatives instead of paraffin wax.^47,48^

Third, all product systems use fabrication methods for rapid prototyping unsuitable for scaling.^7,20,36^ The production methods either become minimally more efficient when scaling up, because large-scale use is inconvenient or illogical, or do not work altogether on a larger scale. Soft lithography in PDMS may be well-suited for rapid prototyping, but its use for commercial-scale production is rare.^49^ This is because PDMS is relatively expensive compared to other materials, and can be fragile once cured.^50,51^ Additionally, soft lithography requires specialized equipment and cleanrooms to develop the molds, further increasing costs.^52^ Achieving high throughput using these techniques is logistically challenging while keeping costs low.^52^ Similarly, for the PLA device, commercial-scale 3D printing is unlikely. The commercial-scale product system was therefore adjusted by assuming injection molding instead. This is a well-established manufacturing technique that has been successfully applied to PLA and is suitable for large-scale production.^53–55^

Across all three products, the use phase is not a significant contributing factor. This is because the use phase does not require any major inputs, outside of electricity in certain cases. Compared to the production and end-of-life, the environmental impacts are therefore lower. For these devices, the main focus should therefore be the manufacturing rather than the use phase. An exception is the commercially produced PLA device. In the contribution analysis, the disk drive is a large contributor in several categories, unlike the laboratory scenario where 3D printing dominated.

Lastly, the reagents and chemicals do not appear to be major contributors to the overall results. This could be due to the model not taking into account water demands and storage requirements. However, since only small volumes of reagents are necessary per device, it is unlikely that including water and storage would significantly affect performance. There are no available studies to compare our findings; more case studies are needed.

### Device Comparability

The PDMS material in the PDMS device is responsible for the higher environmental impact in several categories: ozone depletion, human toxicity: carcinogenic, ecotoxicity: freshwater, and water use. This is because PDMS is based on dimethyldichlorosilane, an inorganic compound used in the synthesis of various silicones.^56^ During its production, many components require cooling, the refrigerants of which are responsible for chlorofluorocarbon emissions, which contribute to ozone depletion.^57,58^ Lastly, the metallurgical-grade silicone required for the production of PDMS is based on coking, the emissions of which typically include carcinogens such as cadmium and arsenic, which have a negative impact on human health.^59^ The high water use is due to the liquid oxygen production, which relies on cryogenic air separation that requires large volumes of cooling water.^60^ Upscaling PDMS production improves efficiency and reduces per-device energy use but has little impact on overall emissions.

The paraffin used for the paper device is responsible for its higher environmental impact in the categories particulate matter formation, energy resources, and material resources. This is because it is a kerosene-based material, and the industrial processes involved in its production are material-intensive and heavily reliant on fossil fuels.^61^ Minimizing paraffin waste and exploring fossil-free alternatives like beeswax or plant-based options could significantly lower emissions for the paper device. The implementation of chitosan improves the color clarity on the device. In literature, some paper devices omit chitosan.^62,63^ We found that chitosan has a negligible impact on overall emissions, enhancing user experience without significantly affecting environmental performance.

The maize grains used for PLA production are responsible for higher impacts in the impact categories of ecotoxicity: freshwater, land use, and water use. This is due to the impacts of maize farming, harvesting, and fertilizer use. The gold in the circuits of the disk drive is responsible for the higher emissions in the category material resources.

The devices all have a similar mode of operation to colorimetrically detect glucose in a sample: through the use of glucose oxidase and a chromogenic agent.^24–26^ There are also differences. First, the proof-of-concept devices target different biofluids: sweat (PDMS), tears (paper), and horse blood plasma (PLA). This results in varying limits of detection. We assumed similar health-monitoring effectiveness across devices, as comparable devices for the same biofluid are rare in literature. However, this overlooks performance differences, which could affect real-world diagnostic accuracy. Adjusting devices for the same biofluid or changing the functional unit could yield different results.

### Upscaling Representativeness

The devices we assess could serve as a viable solution for intermittent, point-of-care screening in settings with limited resources, where the deployment of complex sensor-based continuous glucose monitoring systems may pose logistical or economic challenges. Deployment pathways for these microfluidic devices could include rapid screening in emergency or field settings, use in community-based health campaigns, or veterinary diagnostics, where affordability, disposability, and simplicity are critical considerations.^64,65^

For upscaling production of the PDMS device, we retained the soft lithography method, despite it not being suitable for large-scale production.^50^ This is because there are certain cases where its application has been successful for commercial-scale production of microfluidic devices, such as Standard BioTools with their integrated fluidic circuits for digital PCR.^66,67^ We therefore assumed that a bigger cleanroom would be necessary to facilitate the production of more silicon molds. The increase in cleanroom size is reflected in an increased total electricity consumption, but given that more molds can be produced, the electricity demand per mold actually decreased. Since cleanroom energy use does not scale linearly due to heating, ventilation, and air conditioning (HVAC) and filtration needs, we conducted a sensitivity analysis (Figure S11). Even with nearly double the energy per mold, the commercial-scale PDMS device shows only a slight performance drop, as cleanroom energy contributes minimally to overall impacts. A limitation of the PDMS model is the reliance on expert estimates for silicon mold fabrication due to limited data. This may lead to over- or underestimation of impacts, explaining the mold’s minimal contribution. Using primary data, e.g., from industry, could reduce this uncertainty.

For the paper device, we maintained stamping as a fabrication method when scaling up. While stamping is slow and requires manual operation, some researchers suggest these processes could be scaled for mass production.^68^ This could involve developing a specialized stamping machine to enable automated, assembly line production. This would also lead to less wasted paraffin given the increased efficiency of automated production. Akyazi et al. argue that wax stamping is not suitable for large scale production, as processes for heating the stamp and the immersion of the paper are slow and require too much manual labor.^68^ However, we retained this production method in the upscaled scenario, assuming automation would overcome manual stamping drawbacks. To model this, we used the *deep drawing, steel, 650 kN press, single stroke* ecoinvent process as proxy. We chose this as a suitable proxy due to its similarities and similar level of complexity to the required process, namely heating and stamping. However, as a proxy process, it might not encompass the actual production line that would be required. A more representative production method, such as roll-to-roll printing, might give more insight into an upscaled paper device production process, and would be a recommendation for future studies.

For the PLA device, we found that injection molding is a suitable method to create microfluidic devices, and was a suitable method that one could transition to from 3D printing.^36,69^ We assumed that the geometry of the device had a low enough complexity such that the design could be transferred directly to injection molding. This was modeled using the ecoinvent process for injection molding, and we assumed that the same amount of PLA would be used. This caused a significant decrease in total emissions. However, this modeling choice oversimplifies real-world manufacturing transitions. By not directly modeling the foreground processes, significant flows might be missed. Redesign of the device, such as modifications of the wall thickness or the gate placement, might be necessary to facilitate the process, potentially reducing or increasing environmental impacts depending on material use. Therefore, the actual emissions of the PLA device can potentially be higher than our current estimates. However, our sensitivity analysis (Figure S12) shows that even if material input triples, assuming unchanged performance, the PLA device remains the best-performing alternative. Additionally, we did not account for the material requirements for the mold. This is because they can typically be used millions of times before they need to be replaced.^70^ Therefore, we assumed that the impact of the mold would be negligible. Injection molding provided a straightforward upscaling approach due to its availability in the ecoinvent database. However, alternative methods like hybrid bonding^69^ or laser cutting^71^ could yield different energy and water impacts, potentially altering overall results.

Additionally, we did not include the potential advantages of recycling paper and PLA compared to the limited recyclability of PDMS, as recycling was outside of the scope of this work. Recycling these devices is unlikely due to biological contamination, though end-of-life (EoL) options vary by context. The paper device might be composted with low impact, and PLA has potential for biodegradation, chemical recycling, or industrial composting.^72^ However, these depend on user behavior and local infrastructure, which remains limited. Future research should explore EoL scenarios in greater depth, focusing on how evolving waste systems and improved recycling technologies could affect sustainability, especially at scale.

## Conclusions

We assessed the environmental impact of three microfluidic devices for point-of-care glucose testing, for laboratory and commercial-scale production. We compared soft lithography in PDMS, wax stamping on paper, and 3D-printed PLA. These are all production methods suitable for rapid prototyping in a laboratory setting, using materials that are cheap and widely available. We determined their impacts in the scenario that these devices were pushed to commercial-scale production.

We found that for laboratory-scale production, the wax-stamped paper microfluidic device performed best, due to its simple design and minimal material requirements. The 3D-printed PLA device had the highest environmental impact due to its high energy demand. For commercial-scale production, the PLA device had the lowest environmental impact, while the PDMS device had the highest. This is because we assumed that the PLA device would be produced using injection molding instead of 3D printing for large-scale production. Given that injection molding is a well-established, widely applied technique, the efficiency gains resulted in a large decrease in emissions between production scenarios.

Our findings show that the design of the device and its production method are both important factors when choosing to upscale production. Techniques like soft lithography, manual wax stamping, and 3D printing do not translate well to production scale. While manual wax stamping can be replaced with an automated stamping machine and 3D printing by injection molding, a method like soft lithography is not easily replaced. We found that redesigning was one of the main ways to reduce the environmental impacts of devices, e.g., by investigating alternative materials. Additionally, we found electricity use to be a big contributor, indicating that switching to renewables could reduce impacts. Lastly, we found that the use phase is only a significant contributing factor if the device’s environmental impact is already low. Finally, our assessment is primarily informed by data derived from proof-of-concept papers. Therefore, our results are contingent on scarce primary data and a model largely based on informed estimates and assumptions.

While further research is needed, especially on manufacturing and EoL scenarios, this work offers a comparative assessment of microfluidic devices, highlighting key similarities and differences to guide future development. Moreover, we show the value of applying ex-ante LCA to proof-of-concept technologies, as this can lead to a substantial shift in results compared to laboratory-scale assessments.

## Supplementary Material



## Notes

Additional device details, LCI information, and results, including absolute characterization values (PDF)
