# Supplementary material for: Assessing the Environmental Impacts of Microfluidic Devices for Glucose Detection
Source: ACS Sustain Chem Eng. 2025 Jun 18;13(25):9500–9. doi: 10.1021/acssuschemeng.5c01511 (PMC12216238; doi:10.1021/acssuschemeng.5c01511)
Supplement: Supplementary file 1 [file sc5c01511_si_001.pdf]

Supporting Information

Supporting document for:

Assessing the environmental impacts of microfluidic devices for glucose detection

*Kristie J. Tjokro**†, *Valerio Barbarossa*†‡, *Stefano Cucurachi*†, *Alina Rwei*§, *Justin Lian**†

†Affiliation 1: Institute of Environmental Sciences (CML), Leiden University, Einsteinweg 2, Leiden, 2333 CC, the Netherlands

‡Affiliation 2: Global Sustainability, PBL Netherlands Environmental Assessment Agency, Bezuidenhoutseweg 30, The Hague, 2500 GH, The Netherlands

§ Affiliation 3: Department of Chemical Engineering, Delft University of Technology, Van der Maasweg 9, Delft, 2629 HZ, the Netherlands

* Corresponding author: k.j.tjokro@cml.leidenuniv.nl; z.lian@cml.leidenuniv.nl

Number of pages: 44

Number of tables: 22

Number of figures: 12

Methods

S1 Working principle and manufacture process for microfluidics devices

S1.1 Working principle and manufacture process – PDMS device

Fig. S1 *Process diagram of the manufacturing process for the PDMS device.*

S1.2 Working principle and manufacture process – Paper device

Fig. S2 *Process diagram of the manufacturing process for the paper device.*

S1.3 Working principle and manufacture process – PLA

Fig. S3 *Process diagram of the manufacturing process for the PLA device.*

S2 LCA details

S2.1 Chemicals

Table S1 *An overview of chemicals modelled based on stoichiometry, following the approach of Langhorst et al.*[1].

S2.2 Product system flowcharts

Fig. S4 *Flowchart illustrating the product system of the PDMS microfluidic device designed by Koh et al.* [2].

Fig. S5 *Flowchart illustrating the product system of the paper microfluidic device designed by Gabriel et al.* [3].

Fig. S6 *Flowchart illustrating the product system of the PLA microfluidic device designed by Tothill* [4].

Fig. S7 *Flowchart illustrating the product system of chitosan. This diagram is based on the work of Riofrio et al.*[5].

Fig. S8 *Flowchart illustrating the product system of a medium containing 30,000 units of glucose oxidase, according to Rogalski et al.*[6].

S2.3 Life cycle inventory data

Table S2 *An overview of the inventory tables. Chemical data is given under the alternative they are predominantly used for.*

Table S3 *Inventory data for the product system of the PDMS microfluidic device.*

Table S4 *Inventory data for chemicals required for the preparation of hydrogel for microchannel coating, for the PDMS device.*

Table S5 *Inventory data for chemicals required for the manufacture of the mould for the PDMS device.*

Table S6 *Inventory data for chemicals required for the reagent preparation for the PDMS microfluidic device.*

Table S7 *Inventory data for the product system of the paper microfluidic device.*

Table S8 *Inventory data for chemicals required in the product system of the paper microfluidic device.*

Table S9 *Inventory data for the product system of the PLA microfluidic device.*

Table S10 *Inventory data for unit processes required in multiple product systems.*

Table S11 *Inventory data for glucose oxidase production, which is required in multiple product systems.*

S3 Scaling up production

Table S12 *Differences in magnitudes of economic flows between scenarios for the PDMS device.*

Table S13 *Differences in magnitudes of economic flows between scenarios for the paper device.*

Table S14 *Differences in magnitudes of economic flows between scenarios for the PLA device.*

Results

S4 Characterisation results

S4.1 Characterisation results per scale

Fig. S9 *Characterisation results for the PDMS, paper, and PLA product systems, assuming a laboratory-scale production scenario.*

Fig. S10 *Characterisation results for the PDMS, paper, and PLA product systems, assuming a commercial-scale production scenario.*

Fig. S11 *Sensitivity analysis results for the PDMS device, assuming different production scenarios, cleanroom sizes, and number of molds produced.*

Fig. S12 *Characterisation results for the PDMS, paper, and PLA product systems, for both laboratory and commercial scales. Sensitivity analysis was applied to assess the impact of potentially redesigning the PLA device in a way that would require thrice as much material.*

Table S15 *Characterisation results for the three alternatives: PDMS, paper, and PLA.*

Table S16 *Characterisation results for the PDMS, paper, and PLA device for the case that they are manufactured on a large scale, such as for commercialization.*

S4.2 Contribution analysis

Table S1*7 Contribution analysis for the PDMS device on lab scale, based on the economic flows of the product system. Values shown in percentages.*

Table S18 *Contribution analysis for the PDMS device on commercial scale, based on the economic flows of the product system. Values shown in percentages.*

Table S19 *Contribution analysis for the paper device on lab scale, based on the economic flows of the product system. Values shown in percentages.*

Table S20 *Contribution analysis for the paper device on commercial scale, based on the economic flows of the product system. Values shown in percentages.*

Table S21 *Contribution analysis for the PLA device on lab scale, based on the economic flows of the product system. Values shown in percentages.*

Table S22 *Contribution analysis for the PLA device on commercial scale, based on the economic flows of the product system. Values shown in percentages.*

Materials and Methods

S1 Working principle and manufacture process for microfluidics devices

S1.1 Working principle and manufacture process – PDMS device

Based on the work of Koh et al[2]. The PDMS device detects glucose, lactate, and chloride ions in sweat, and gives an indication of the pH of the sweat. For detecting glucose in human sweat, the obtained LOD was 0.2 mM.

The device is wearable and sticks to the skin by means of an adhesive layer. Once the wearer starts perspiring, the perspiration flows through the micro-channels until it reaches the assays. Upon reaction, the fluid changes colour, thereby giving a colorimetric indication of the glucose, lactate, chloride ions and pH levels of the wearer's sweat.

While the colorimetric results give indications of biomarker levels that are visible to the naked eye, the results can be quantified and analysed even further using NFC electronics and a smartphone. Upon scanning the device, its NFC chip will launch a program for image processing on the user's smartphone. The software then provides the user with the RGB values that the device's colouring has taken on. Using these values, the biomarker levels can be quantified.

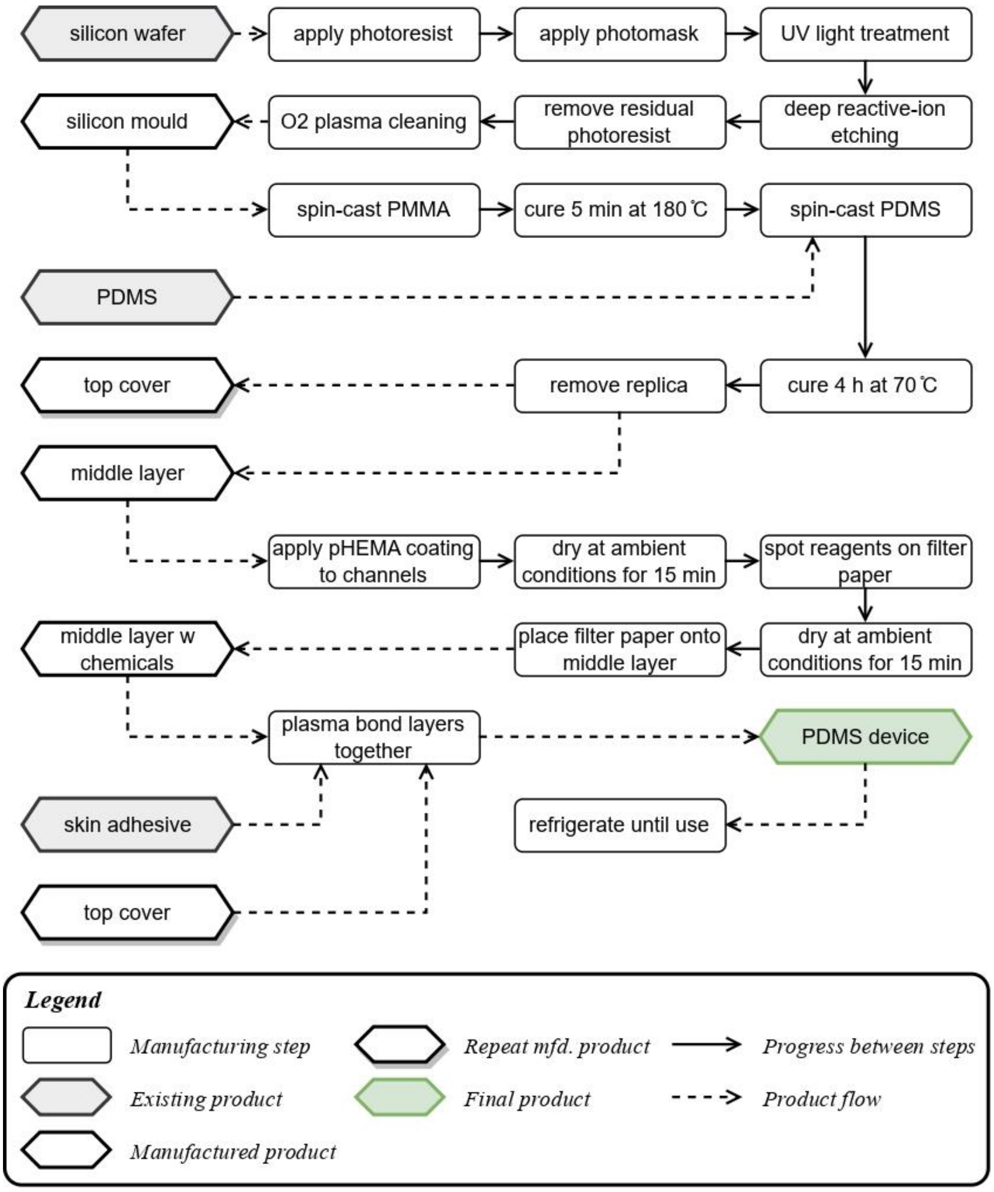

Fig. S1 *Process diagram of the manufacturing process for the PDMS device. The top cover and middle layer are manufactured using the same process, while the skin adhesive is purchased from a third party. Once the PDMS device has been completed, it must be refrigerated until use to preserve the inserted enzymes.*

S1.2 Working principle and manufacture process – Paper device

Based on the work of Gabriel et al.[3], the device detects glucose in the sample and changes colour based on the glucose concentration. For detecting glucose in human tears, the obtained LOD was 32µM. Paper-based microfluidic devices for colorimetric detection often suffer from poor colour uniformity, which impairs the analytical reliability [3]. The authors show that the quality of the colorimetric measurements can be improved using chitosan. The chitosan application modifies the surface and creates a micro-environment where direct electron transfer between enzyme and reagent can take place more effectively. Comparing the chitosan-enriched paper device to standard µPADs, there is a noticeable improvement in colour uniformity and pixel intensity in the detection zones.

The paper device is used for glucose detection by depositing a human sample onto the central zone – in this case human tears are used. Once the sample is applied, the capillary forces present in the wet paper force the tears to flow into the detection zones, prevented by the wax from flowing outside of the barriers. Once the colour in the detection zone has changed, the paper device can be scanned and assigned RGB values. The intensity and gradient of the colour are then used to quantify glucose levels.

The paper devices were fabricated as follows:

Preheat stainless steel stamp to 150°C.

Impregnate sheet of filter paper with liquid paraffin wax and allow it to dry.

Place native filter paper on top of impregnated paper.

Once the stamp is hot, place the stamp on layered paper.

Place pressure onto the stamp for 2 s to allow paraffin to melt onto the native paper and form hydrophobic barriers.

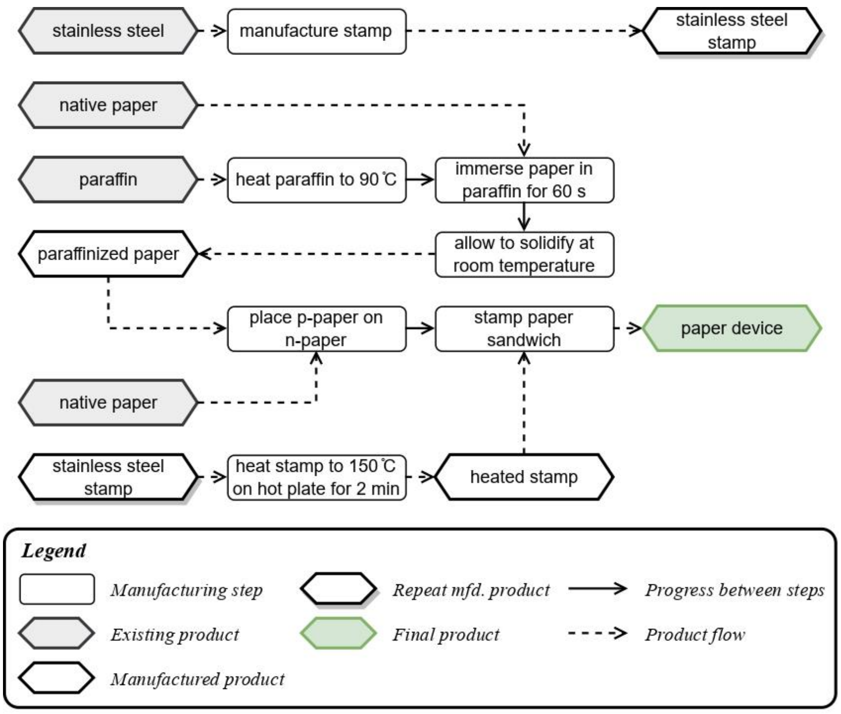

Fig. S2 *Process diagram of the manufacturing process for the paper device. By placing native paper on top of paraffin-impregnated paper, and stamping this with a hot stamp, hydrophobic wax barriers are formed.*

S1.3 Working principle and manufacture process - PLA

Based on the work of Tothill[4]. For glucose detection, a blood sample is mixed with a glucose assay. The glucose assay consists of chromotropic acid, 4-aminoantipyrine, horseradish peroxidase, and glucose oxidase. Upon mixing, the sample-assay mixture is injected into the device's channels. The PLA device is placed onto an optical disk drive (ODD) for centrifugation to force blood separation. Once centrifugation is done, a distinct colour can

be seen on the device where the plasma has reacted with the glucose assay. For detecting glucose in blood plasma, the obtained LOD was 0.6 mM.

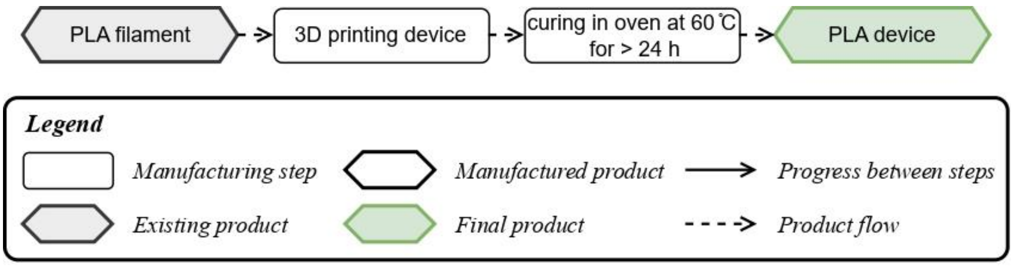

Fig. S3 *Process diagram of the manufacturing process for the PLA device. Using PLA filament, the device is 3D printed. To ensure the device is leakproof, it undergoes heat treatment in an electric convection oven for over 24h.*

S2 LCA details

S2.1 Chemicals

The microfluidic devices of this work all require a range of chemicals to detect glucose in samples. While some chemicals are available in the ecoinvent database, those that are not will be modelled based on literature or stoichiometry. The stoichiometry-based modelling was done according to the method described by Langhorst et al.[1].

In the case of missing LCI data for chemicals, the approach described by Huber et al.[7] was used. The authors provide recommendations for which method to use depending on the situation. When modelling the inventory data for a chemical that is unavailable in LCA datasets, a proxy can be found in a different chemical that is sufficiently similar. When there is no suitable proxy, the inventory data can be based on the stoichiometry, or chemical balance, for the synthesis of the chemical.

Langhorst et al. provide a method for stoichiometry-based estimation of chemical data. Their method is based on various other methods[8–12]. These models have different values for the yield and have different assumptions regarding energy and water consumption of synthesis processes. By comparing models and aggregating the best aspects between them, Langhorst et al. propose a combined estimation method. Here, they chose the approach of Geisler et al., who assume a yield of 87% when there are no significant by-products (like water), and a yield of 77% if by-products may occur. They also chose the approach of Kim & Overcash, who provide average values for the energy demands of the synthesis processes.

Primary data was collected for the electricity consumption of an electric convection oven. Given the cycles of heating and cooling an oven experience during its operation, it would not be possible to ensure the accuracy of energy calculations without knowing the specifications of the cycles. While this cannot be ensured for any energy calculations, it was especially necessary for the oven, given that the oven is used for more than 24 h to cure the PLA device. Therefore, data was collected by measuring the electricity consumption of an oven in a soft lithography laboratory. The oven was kept at a constant temperature of 70 °C; it was measured over the span of a weekend, for more than 80 h.

The microfluidic devices of this study require various chemicals to function. Some of these could be found in the ecoinvent database, as introduced above, while others were unavailable. This latter group of chemicals was

therefore modelled manually based on data from literature. A distinction is made between chemicals that were modelled based on stoichiometry alone (listed in Table S1), and those that were modelled based on similar studies.

Table S1 *An overview of chemicals modelled based on stoichiometry, following the approach of* [1].

Chemical name	Chemical formula	Chemical equation
**ammonium persulfate**	$(NH_4)_2S_2O_8$	Equation A.1
**cobalt(ii)chloride**	$CoCl_2$	Equation A.2
**dipotassium monophosphate**	K_2HPO_4	Equation A.3
**ethylene glycol dimethacrylate**	$C_{10}H_{14}O_4$	Equation A.4
**ethylene glycol monomethacrylate**	$C_6H_{10}O_3$	Equation A.5
**monopotassium diphosphate**	KH_2PO_4	Equation A.6
**sodium thiosulfate**	$Na_2S_2O_3$	Equation A.7
**tetramethylammonium chloride**	$C_4H_{12}NCl$	Equation A.8
**tetramethylammonium hydroxide**	$C_4H_{13}NO$	Equation A.9
**trisodium citrate**	$Na_3C_6H_5O_7$	Equation A.10

Potassium iodide is modelled based on Gong et al.[13], who conducted an LCA of perovskite photovoltaics and provided detailed unit process data. Magnesium hydroxide production was modelled after the ecoinvent process “salt production from seawater, evaporation pond", and was expanded on by using information provided by Seeger et al.[14]. Here, the authors describe the process of precipitating magnesium hydroxide from seawater, which was sufficiently similar to the ecoinvent process. Trisodium citrate production is based on Yao et al[15].

Ammonium sulfate + Sulfuric acid → Ammonium persulfate + hydrogen gas

$$(NH_4)_2SO_4 + H_2SO_4 \rightarrow (NH_4)_2S_2O_8 + H_2 \quad (A.1)$$

Cobalt hydroxide + Hydrochloric acid → Cobalt(ii)chloride

$$Co(OH)_2 + 2\ HCl \rightarrow CoCl_2 \quad (A.2)$$

Phosphoric acid + Potassium hydroxide → Dipotassium monophosphate

$$H_3PO_4 + 2\ KOH \rightarrow K_2HPO_4 \quad (A.3)$$

Methacrylic acid + Ethylene glycol → Ethylene glycol dimethacrylate

$$2\ C_5H_8O_2 + C_2H_6O_2 \rightarrow C_{10}H_{14}O_4 \quad (A.4)$$

Phosphoric acid + Potassium chloride → Monopotassium diphosphate + Hydrochloric acid

$$H_3PO_4 + KCl \rightarrow KH_2PO_4 + HCl \quad (A.5)$$

Sodium sulfide + Sulfur → Sodium thiosulfate

$$Na_2SO_3 + S \rightarrow Na_2S_2O_3 \quad (A.7)$$

Trimethylamine + Methylchloride → Tetramethylammonium chloride

$$C_3H_9N + CH_3Cl \rightarrow C_4H_{12}NCl \quad (A.8)$$

Tetramethylammonium chloride + Potassium hydroxide
→ Tetramethylammonium hydroxide + Potassium chloride

$$C_4H_{12}NCl + KOH \rightarrow C_4H_{13}NO + KCl \quad (A.9)$$

Citric acid + Sodium hydroxide → Trisodium citrate

$$C_6H_8O_7 + 3\ NaOH \rightarrow Na_3C_6H_5O_7 \quad (A.10)$$

S2.2 Product system flowcharts

For the flowcharts, some processes have been omitted for clarity. In the case of the preparation of chemical solutions in water, the intermediate step of diluting the chemical in water has been left out. Instead, the addition of water is added to the main chemical production step, which then continues directly to the unit process the solution is used in. For example, in the PDMS flowchart, when a sodium thiosulfate solution is required, the input of water occurs in the unit process to produce sodium thiosulfate. The output then flows directly to pHEMA hydrogel preparation, instead of an intermediate process called "sodium thiosulfate solution preparation".

This is inconsistent with the LCA model, where these intermittent steps were modelled separately for ease of calculation. However, in the flowchart, adding these processes would only clutter the diagram, while not contributing to making the system more understandable. This must be taken into consideration when assessing the flowchart and comparing it to the inventory data tables.

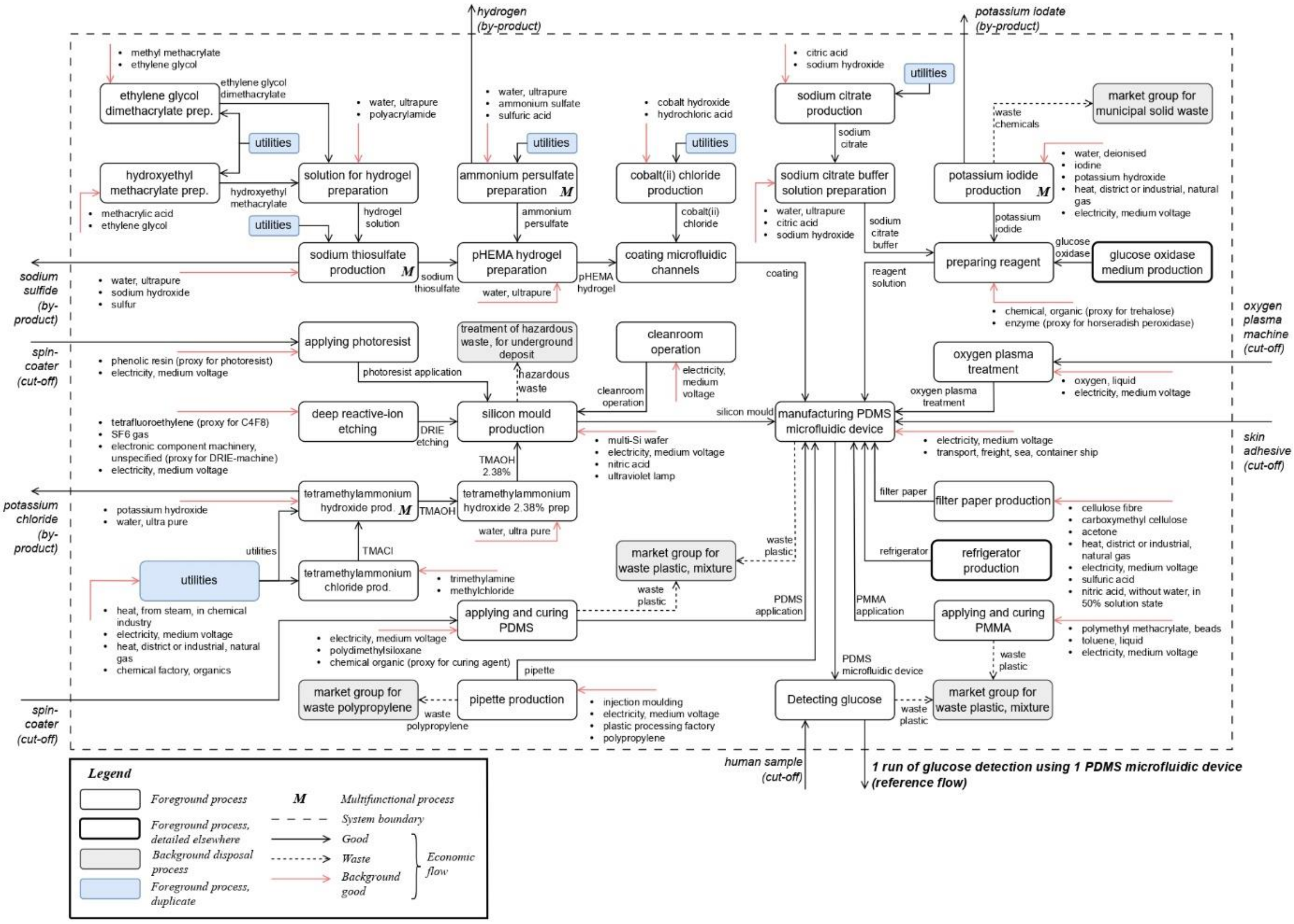

Fig. S4 *Flowchart illustrating the product system of the PDMS microfluidic device designed by Koh et al.*[2]*. This diagram has been created based on additional information from personal communication with an expert of soft lithography.*

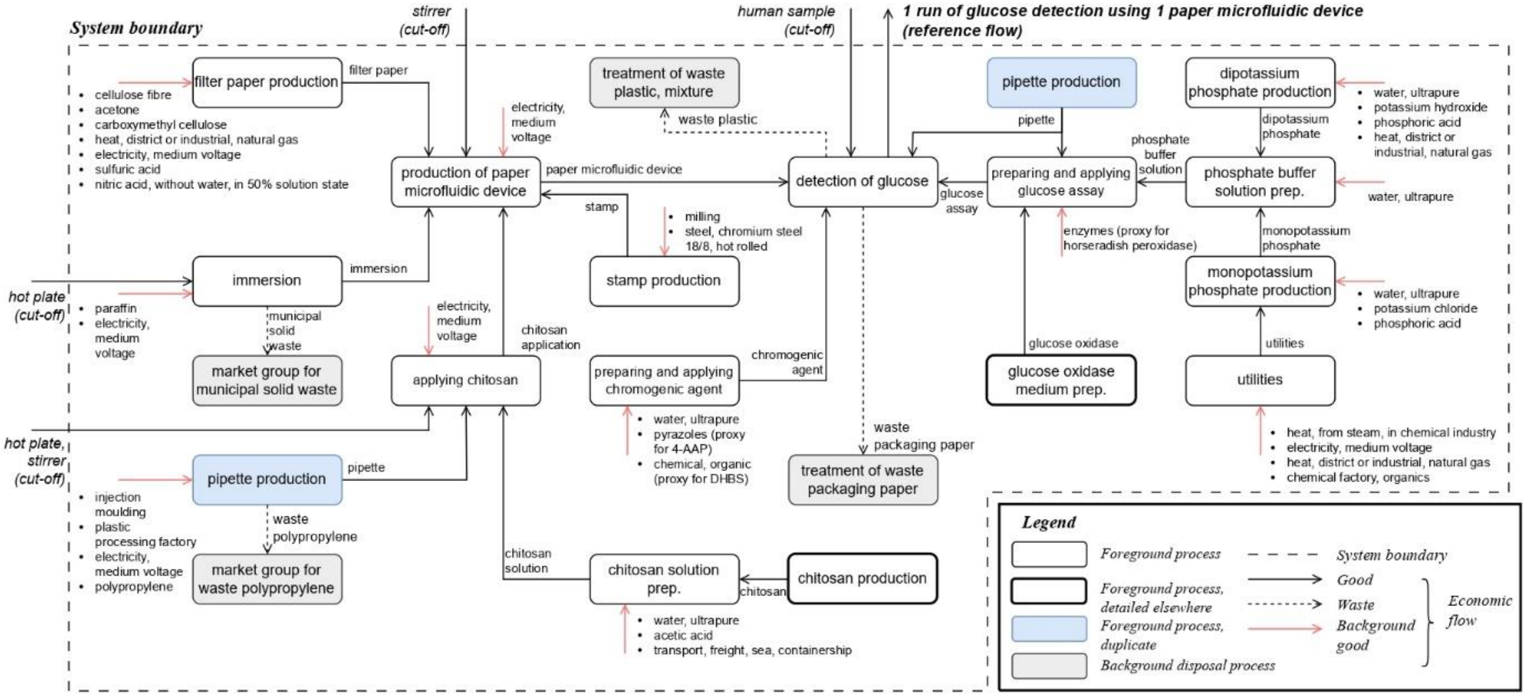

Fig. S5 *Flowchart illustrating the product system of the paper microfluidic device designed by Gabriel et al.*[3].

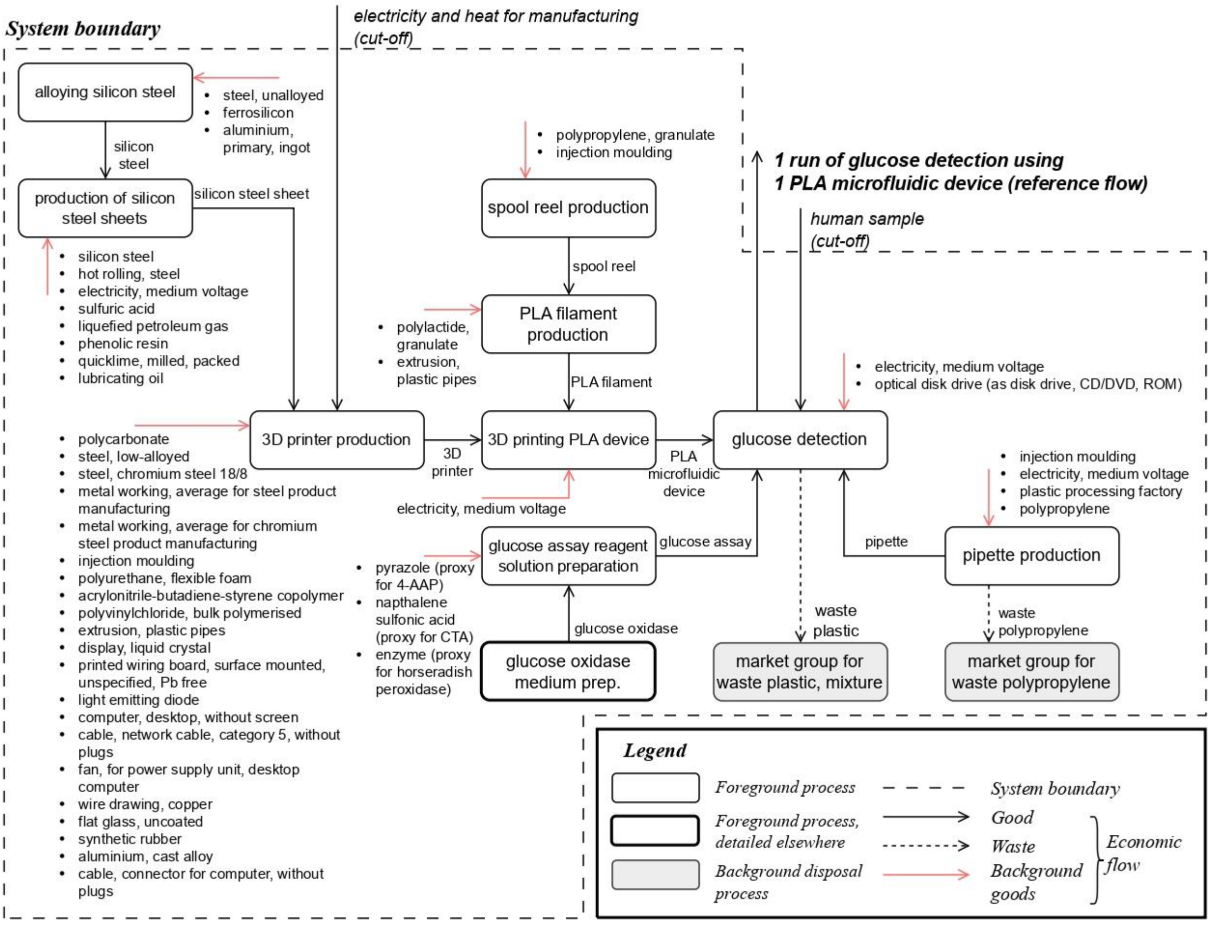

Fig. S6 *Flowchart illustrating the product system of the PLA microfluidic device designed by Tothill*[4].

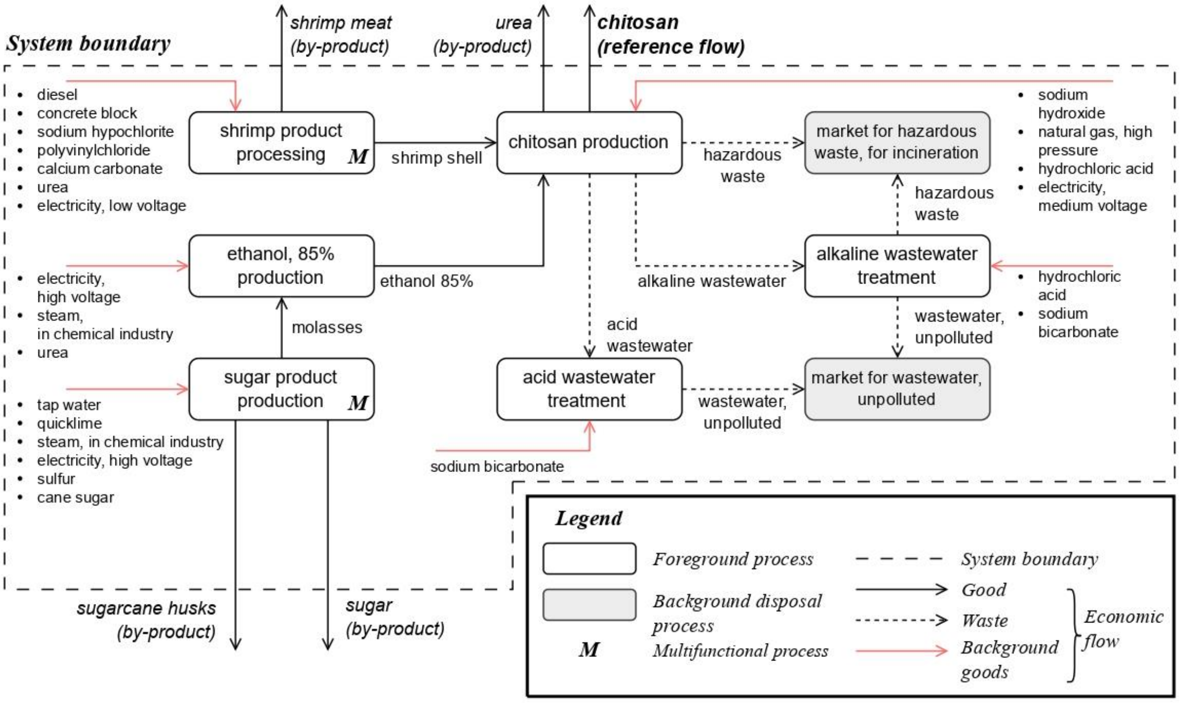

Fig. S7 *Flowchart illustrating the product system of chitosan. This diagram is based on the work of Riofrio et al.*[5].

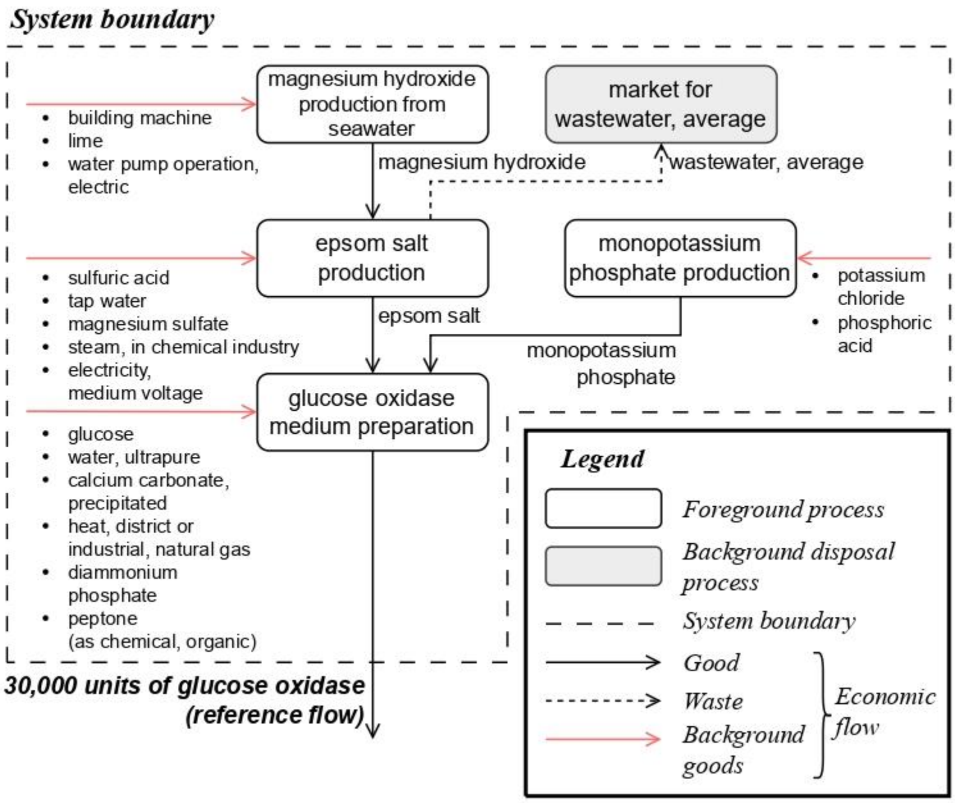

Fig. S8 *Flowchart illustrating the product system of a medium containing 30,000 units of glucose oxidase, according to Rogalski et al.*[6].

S2.3 Life cycle inventory data

An overview of the inventory tables provided in Tables below.

Table S2 *An overview of the inventory tables. Chemical data is given under the alternative they are predominantly used for.*

System	Inventory table number
**PDMS alternative**	Table S3
**Chemicals for coating PDMS microchannels**	Table S4
**Chemicals for manufacturing PDMS mould**	Table S5
**Chemicals for reagent, PDMS**	Table S6
**Paper alternative**	Table S7
**Chemicals for glucose assay, paper**	Table S8
**PLA alternative**	Table S9
**Glucose oxidase**	Table S10
**Duplicate processes**	Table S11

Table S3 *Inventory data for the product system of the PDMS microfluidic device.*

Flow name	Quantity	Unit
Applying photoresist[2]		
Economic inputs		
electricity, medium voltage	1.50E-02	kilowatt hour
phenolic resin (proxy for photoresist)	1.41E-02	kilogram
Economic outputs		
photoresist application	1.00E+00	unit
Cleanroom operations[16]		
Economic inputs		
electricity, medium voltage	6.73E+00	kilowatt hour
Economic outputs		
cleanroom operations	1.00E+00	unit
Deep reactive-ion etching[2]		
Economic inputs		

electricity, medium voltage	1.67E+00	kilowatt hour
sulfur hexafluoride, liquid	7.51E-02	kilogram
tetrafluoroethylene (proxy for C4F8)	2.36E-02	kilogram
Economic outputs		
deep-reactive ion etching	1.00E+00	unit
Silicon mold production[2].		
Economic inputs		
photoresist application	1.00E+00	unit
deep-reactive ion etching	1.00E+00	unit
cleanroom operations	1.00E+00	unit
tetramethylammonium hydroxide 2.38% soln	4.00E-01	liter
multi-Si wafer	7.85E-03	square meter
nitric acid, without water, in 50% soln state	6.04E-04	kilogram
electricity, medium voltage	6.67E-05	kilowatt hour
electronic component machinery, unspecified	1.60E-05	unit
ultraviolet lamp	9.26E-07	unit
Economic outputs		
silicon mould	1.00E+00	unit
hazardous waste, for underground deposit	3.99E-01	kilogram
Oxygen plasma treatment[2]		
Economic inputs		
oxygen, liquid	8.57E-01	kilogram
Electricity, medium voltage	3.75E-02	kilowatt hour
Economic outputs		
oxygen plasma treatment	1.00E+00	unit
Applying and curing PMMA[2]		
Economic inputs		
electricity, medium voltage	4.80E-03	kilowatt hour

toluene, liquid	4.17E-09	kilogram
polymethyl methacrylate, beads	2.20E-10	kilogram
Economic outputs		
PMMA application	1.00E+00	unit
waste plastic, mixture	3.95E-09	kilogram
Applying and curing PDMS[2]		
Economic inputs		
electricity, medium voltage	7.70E-02	kilowatt hour
polydimethylsiloxane	4.83E-03	kilogram
Economic outputs		
PDMS application	1.00E+00	unit
waste plastic, mixture	4.34E-03	kilogram
Coating microfluidic channels[2]		
Economic inputs		
pHEMA hydrogel	1.00E+00	liter
cobalt(ii) chloride	1.00E-01	kilogram
Economic outputs		
coating for microfluidic channels	1.00E+00	liter
Preparing reagent for PDMS device[2]		
Economic inputs		
glucose oxidase	1.20E+05	unit
sodium citrate buffer solution	1.00E+00	liter
chemical, organic	1.02E-01	kilogram
potassium iodide	9.96E-02	kilogram
enzymes	1.20E-04	kilogram
Economic outputs		
reagent for PDMS device	1.00E+00	liter
Manufacturing PDMS microfluidic device[2]		

Economic inputs		
PMMA application	2.00E+00	unit
PDMS application	1.29E+00	unit
oxygen plasma treatment	1.00E+00	unit
electricity, medium voltage	3.10E-01	kilowatt hour
transport, freight, sea, container ship	3.25E-02	ton kilometer
silicon mould	4.00E-03	unit
pipette	2.20E-03	kilogram
coating for microfluidic channels	5.00E-05	liter
reagent for PDMS device	5.00E-06	liter
filter paper	1.01E-06	kilogram
Economic outputs		
PDMS microfluidic device	1.00E+00	unit
waste plastic, mixture	1.38E-03	kilogram
Detecting glucose using PDMS device[2]		
Economic inputs		
PDMS microfluidic device	1.00E+00	unit
Economic outputs		
run of detecting glucose using PDMS device	1.00E+00	unit
waste plastic, mixture	6.18E-04	kilogram

Table S4 *Inventory data for chemicals required for the preparation of hydrogel for microchannel coating, for the PDMS device.*

Flow name	Quantity	Unit
Ethylene glycol dimethacrylate production		
Economic inputs		
utilities	1.00E+00	unit
methacrylic acid	9.98E-01	kilogram
ethylene glycol	3.60E-01	kilogram

Economic outputs		
ethylene glycol dimethacrylate	1.00E+00	kilogram
Hydroxyethyl methacrylate production[17]		
Economic inputs		
ethylene glycol	5.48E-01	kilogram
utilities	1.00E+00	unit
methacrylic acid	0.76E-01	kilogram
Economic outputs		
hydroxyethyl methacrylate	1.00E+00	kilogram
Solution for hydrogel preparation[18]		
Economic inputs		
water, ultrapure	7.67E-01	kilogram
polyacrylamide	1.54E-01	kilogram
hydroxyethyl methacrylate	1.02E-01	kilogram
ethylene glycol dimethacrylate	1.03E-03	kilogram
Economic outputs		
solution for hydrogel	1.00E+00	liter
Ammonium persulfate production[19]		
Economic inputs		
utilities	1.00E+00	unit
ammonium sulfate	7.52E-01	kilogram
sulfuric acid	5.58E-01	kilogram
Economic outputs		
ammonium persulfate	1.00E+00	kilogram
hydrogen	8.84E-03	kilogram
Ammonium persulfate solution production[2]		
Economic inputs		
water, ultrapure	9.80E-01	kilogram

ammonium persulfate	2.00E-02	kilogram
Economic outputs		
ammonium persulfate solution	1.00E+00	liter
Sodium thiosulfate production[20]		
Economic inputs		
utilities	1.00E+00	unit
sodium sulfite	9.16E-01	kilogram
sulfur	2.33E-01	kilogram
Economic outputs		
sodium thiosulfate	1.00E+00	kilogram
Sodium thiosulfate solution production[2]		
Economic inputs		
water, ultrapure	9.98E-01	kilogram
sodium thiosulfate	2.00E-03	kilogram
Economic outputs		
sodium thiosulfate solution	1.00E+00	liter
pHEMA production[18]		
Economic inputs		
solution for hydrogel	4.11E+00	liter
ammonium persulfate solution	6.16E-02	liter
sodium thiosulfate solution	4.11E-02	liter
Economic outputs		
pHEMA	1.00E+00	kilogram
pHEMA hydrogel production[18]		
Economic inputs		
water, ultrapure	9.98E-01	kilogram
pHEMA	2.00E-03	kilogram
Economic outputs		

pHEMA hydrogel	1.00E+00	kilogram
Cobalt(ii) chloride production[21]		
Economic inputs		
utilities	1.00E+00	unit
cobalt hydroxide	8.23E-01	kilogram
hydrochloric acid, w/o water, in 30% soln state	6.46E-01	kilogram
Economic outputs		
cobalt(ii) chloride	1.00E+00	kilogram

Table S5 *Inventory data for chemicals required for the manufacture of the mold for the PDMS device.*

Flow name	Quantity	Unit
Tetramethylammonium chloride production[22]		
Economic inputs		
utilities	1.00E+00	unit
trimethylamine	6.20E-01	kilogram
methylchloride	5.30E-01	kilogram
Economic outputs		
tetramethylammonium chloride	1.00E+00	kilogram
Tetramethylammonium hydroxide production[23]		
Economic inputs		
tetramethylammonium chloride	1.56E+00	kilogram
utilities	1.00E+00	unit
potassium hydroxide	7.99E-01	kilogram
Economic outputs		
tetramethylammonium hydroxide	1.00E+00	kilogram
potassium chloride	8.18E-01	kilogram
Tetramethylammonium hydroxide 2.38% preparation[2]		
Economic inputs		

water, ultrapure	9.73E-01	kilogram
tetramethylammonium hydroxide	2.37E-02	kilogram
Economic outputs		
tetramethylammonium hydroxide 2.38% soln	1.00E+00	liter

Table S6 *Inventory data for chemicals required for the reagent preparation for the PDMS microfluidic device.*

Flow name	Quantity	Unit
Sodium citrate production[15]		
Economic inputs		
utilities	1.00E+00	unit
citric acid	8.56E-01	kilogram
sodium hydroxide, w/o water, in 50% soln state	5.34E-01	kilogram
Economic outputs		
sodium citrate	1.00E+00	kilogram
Sodium citrate solution preparation[2]		
Economic inputs		
water, ultrapure	1.00E+00	kilogram
sodium citrate	2.94E-02	kilogram
Economic outputs		
sodium citrate solution	1.00E+00	kilogram
Citric acid solution preparation[2]		
Economic inputs		
water, ultrapure	9.97E-01	kilogram
citric acid	2.10E-02	kilogram
Economic outputs		
citric acid solution	1.00E+00	liter
Sodium hydroxide 1M preparation[2]		
Economic inputs		

water, ultrapure	1.00E+00	kilogram
sodium hydroxide, w/o water, in 50% soln state	4.00E-02	kilogram
Economic outputs		
sodium hydroxide 1M	1.00E+00	liter
Sodium citrate buffer solution production		
Economic inputs		
water, ultrapure	8.90E-01	kilogram
citric acid solution	8.20E-02	liter
sodium citrate solution	1.80E-02	liter
sodium hydroxide 1M	1.00E-02	liter
Economic outputs		
sodium citrate buffer solution	1.00E+00	liter
Potassium iodide production[13]		
Economic inputs		
heat, district or industrial, natural gas	1.72E+00	megajoule
iodine	9.33E-01	kilogram
water, deionised	8.00E-01	kilogram
potassium hydroxide	4.05E-01	kilogram
electricity, medium voltage	1.19E-02	kilowatt hour
Economic outputs		
potassium iodide	1.00E+00	kilogram
potassium iodate	1.88E-01	kilogram
municipal solid waste	1.35E-02	kilogram

Table S7 *Inventory data for the product system of the paper microfluidic device.*

Flow name	Quantity	Unit
Stamp production[24]		
Economic inputs		

steel, chromium steel 18/8, hot rolled	7.18E-02	kilogram
chromium steel removed by milling, small parts	2.15E-02	kilogram
Economic outputs		
stamp	1.00E+00	unit
Immersion process for 1 paper device[24]		
Economic inputs		
paraffin	4.66E-01	kilogram
electricity, medium voltage	1.35E-01	kilogram
Economic outputs		
immersion for 1 paper device	1.00E+00	unit
municipal solid waste	2.33E-01	kilogram
Chitosan in acetic acid solution preparation[3]		
Economic inputs		
water, ultrapure	9.77E-01	kilogram
electricity, medium voltage	3.00E-01	kilowatt hour
transport, freight, sea, container ship	7.11E-02	ton kilometre
acetic acid, without water, in 98% solution state	2.10E-02	kilogram
chitosan	5.00E-03	kilogram
Economic outputs		
chitosan solution	1.00E+00	litre
Applying chitosan solution[3]		
Economic inputs		
pipette	1.10E-03	kilogram
chitosan solution	3.00E-06	litre
Economic outputs		
chitosan application for 1 paper device	1.00E+00	unit
Manufacturing paper microfluidic device[3,24]		
Economic inputs		

immersion	1.00E+00	unit
chitosan application	1.00E+00	unit
filter paper	1.96E-03	kilogram
electricity, medium voltage	1.30E-03	unit
stamp	2.00E-04	kilowatt hour
Economic outputs		
paper microfluidic device	1.00E+00	unit
Preparing reagent for paper device[3]		
Economic inputs		
water, ultrapure	1.00E+00	kilogram
chemical, organic (proxy for DHBS)	2.12E-03	kilogram
pyrazole (proxy for 4-AAP)	8.13E-04	kilogram
Economic outputs		
reagent solution for paper device	1.00E+00	liter
Preparing glucose assay for paper device [3]		
Economic inputs		
glucose oxidase	1.20E+05	unit
phosphate buffer solution 0.1M pH6.0	1.00E+00	liter
enzymes (proxy for horseradish peroxidase)	1.20E-04	kilogram
Economic outputs		
glucose assay solution for paper device	1.00E+00	liter
Detecting glucose using paper device [3]		
Economic inputs		
paper microfluidic device	1.00E+00	unit
pipette	3.30E-03	kilogram
reagent solution for paper device	1.00E-06	liter
glucose assay for paper device	1.00E-06	liter
Economic outputs		

run of detecting glucose using paper device	1.00E+00	unit
waste plastic, mixture	3.30E-03	kilogram
waste packaging paper	3.62E-04	kilogram

Table S8 *Inventory data for chemicals required in the product system of the paper microfluidic device.*

Flow name	Quantity	Unit
Monopotassium diphosphate production[25]		
Economic inputs		
utilities	1.00E+00	unit
phosphoric acid, industrial grade, w/o water, in 85% soln state	9.35E-01	kilogram
potassium chloride	7.11E-01	kilogram
Economic outputs		
monopotassium diphosphate	1.00E+00	kilogram
hydrochloric acid	2.68E-01	kilogram
Monopotassium phosphate, 1M solution preparation[3]		
Economic inputs		
water, ultrapure	9.98E-01	kilogram
monopotassium diphosphate	1.36E-01	kilogram
Economic outputs		
monopotassium phosphate, 1M solution	1.00E+00	liter
Dipotassium phosphate production[25]		
Economic inputs		
utilities	1.00E+00	unit
potassium hydroxide	7.40E-01	kilogram
phosphoric acid, industrial grade, w/o water, in 85% soln state	6.47E-01	kilogram
Economic outputs		
dipotassium phosphate	1.00E+00	kilogram
Dipotassium phosphate, 1M solution preparation[3]		

Economic inputs		
water, ultrapure	9.98E-01	kilogram
dipotassium phosphate	1.74E-01	kilogram
Economic outputs		
dipotassium phosphate, 1M solution	1.00E+00	liter
Phosphate buffer solution production 0.1M pH 6.0[3,26]		
Economic inputs		
water, ultrapure	8.97E-01	kilogram
monopotassium phosphate, 1M solution	8.68E-02	liter
dipotassium phosphate, 1M solution	1.32E-02	liter
Economic outputs		
phosphate buffer solution 0.1M pH 6.0	1.00E+00	liter

Table S9 *Inventory data for the product system of the PLA microfluidic device.*

Flow name	Quantity	Unit
Spool reel production[27]		
Economic inputs		
polypropylene, granulate	1.00E+00	kilogram
injection moulding	1.00E+00	kilogram
Economic outputs		
spool reel	1.00E+00	kilogram
PLA filament production[27]		
Economic inputs		
extrusion, plastic pipes	7.53E-01	kilogram
polylactide, granulate	7.53E-01	kilogram
spool reel	2.50E-01	kilogram
Economic outputs		
PLA filament	1.00E+00	kilogram

3D printing PLA microfluidic device[4]		
Economic inputs		
electricity, medium voltage, from scenario	6.32E+00	kilowatt hour
PLA filament	1.40E-02	kilogram
Economic outputs		
PLA microfluidic device	1.00E+00	unit
Preparing glucose assay reagent solution for PLA device[4]		
Economic inputs		
glucose oxidase	2.00E+04	unit
naphthalene sulfonic acid (proxy for CTA)	1.28E-02	kilogram
pyrazole (proxy for 4-AAP)	4.06E-03	kilogram
enzyme (proxy for horseradish peroxidase)	2.40E-05	kilogram
Economic outputs		
glucose assay reagent solution for PLA device	1.00E+00	litre
Detecting glucose using PLA device[4]		
Economic inputs		
PLA microfluidic device	1.00E+00	unit
pipette	1.10E-03	kilogram
electricity, medium voltage, from scenario	3.83E-04	kilowatt hour
disk drive, CD/DVD, ROM, for desktop computer	3.70E-04	unit
glucose assay reagent solution for PLA device	9.20E-05	litre
Economic outputs		
run of detecting glucose using PLA device	1.00E+00	unit
municipal solid waste	1.51E-02	kilogram

Table S10 *Inventory data for unit processes required in multiple product systems.*

Flow name	Quantity	Unit
Filter paper production[28]		

Economic inputs		
heat, district or industrial, natural gas	5.00E+01	megajoule
sulfuric acid	1.27E+00	kilogram
cellulose fibre	9.47E-01	kilogram
acetone, liquid	7.85E-01	kilogram
nitric acid, w/o water, in 50\% soln state	6.36E-01	kilogram
electricity, medium voltage	1.51E-01	kilowatt hour
carboxymethyl cellulose, powder	6.98E-03	kilogram
Economic outputs		
filter paper	1.00	kilogram
Pipette production		
Economic inputs		
polypropylene, granulate	1.00E+00	kilogram
injection moulding	1.00E+00	kilogram
plastic processing factory	7.40E-10	unit
Economic outputs		
pipette	1.00E+00	kilogram
Utilities[1]		
Economic inputs		
heat, from steam, in chemical industry	6.10E+00	megajoule
electricity, medium voltage	1.67E-01	kilowatt hour
heat, district or industrial, natural gas	1.50E-01	megajoule
chemical factory, organics	4.00E-10	unit
Economic outputs		
utilities	1.00E+00	unit

Table S11 *Inventory data for glucose oxidase production, which is required in multiple product systems.*

Flow name	Quantity	Unit

Magnesium hydroxide production from seawater		
Economic inputs		
lime	1.27E+00	kilogram
water pump operation, electric	1.00E-03	megajoule
building machine	1.00E-08	unit
Economic outputs		
magnesium hydroxide	1.00E+00	kilogram
Environmental inputs		
Occupation, seabed, unspecified (natural resource - land)	7.32E+04	square meter-year
Water, salt, sole (natural resource - in water)	3.20E+03	cubic meter
Epsom salt production		
Economic inputs		
electricity, medium voltage	1.31E+01	kilowatt hour
steam, in chemical industry	7.48E+00	kilogram
tap water	1.00E+00	kilogram
magnesium hydroxide	4.00E-01	kilogram
sulfuric acid	3.70E-01	kilogram
magnesium sulfate	9.00E-02	kilogram
Economic outputs		
epsom salt production	1.00E+00	kilogram
wastewater, average	1.17E-03	cubic meter
Environmental inputs		
Water, unspecified natural origin	5.78E-02	cubic meter
Glucose oxidase medium production		
Economic inputs		
water, ultrapure	1.00E+00	kilogram
glucose	8.00E-02	kilogram
heat, district or industrial, natural gas	5.40E-02	megajoule

calcium carbonate, precipitated	3.50E-02	kilogram
chemical, organic	3.00E-02	kilogram
diammonium phosphate	3.88E-04	kilogram
monopotassium diphosphate	1.88E-04	kilogram
epsom salt production	1.56E-04	kilogram
Economic outputs		
glucose oxidase	3.00E+04	unit

S3 Scaling up production

PDMS

From an industrial standpoint, manufacturing PDMS devices is expensive. There are a few examples of successful PDMS devices made through soft lithography, such as those sold by Fluidigm [29], but generally manufacturers prefer thermoplastics, glass, and silicon over PDMS. Taking Fluidigm as an example, the modelling of the commercial-scale LCA, little was changed to the manufacturing processes of the PDMS device. Improvements of efficiencies were achieved by reducing the space or electricity required per device produced. This was done by reducing the amount of cleanroom space required per mould produced. Another adjustment is that the curing of PDMS and PMMA is now performed in an electric oven, with space for 10 wafers to be cured simultaneously. This oven is modelled based on the oven used in the product system of the PLA device. The changes in economic flows between the models for laboratory-scale and commercial-scale are shown in Table S12.

Table S12 *Differences in magnitudes of economic flows between scenarios for the PDMS device.*

Flow name	Laboratory	Commercial	Unit
[A1.2] Cleanroom operations			
Economic inputs			
electricity, medium voltage	6.73E+00	1.68E+00	kilowatt hour
[A1.7] Applying and curing PDMS			
Economic inputs			
electricity, medium voltage	7.70E-02	3.85E-03	kilowatt hour
[A1.6] Applying and curing PMMA			
Economic inputs			
electricity, medium voltage	4.80E-03	2.41E-04	kilowatt hour
PDMS microfluidic device manufacturing			
Economic inputs			
electricity, medium voltage	3.10E-02	1.55E-03	kilowatt hour

Paper

As discussed by Akyazi et al. (2018), wax stamping is an inefficient manufacturing method that is unsuitable for mass production. While it is ideal for field research, and in low resource settings, the processes for heating the stamp and the immersion of the paper are slow and require too much manual labour. Nevertheless, the method was retained to model large-scale production.

In the model, the amount of paraffin wax that is wasted is reduced significantly. As more pieces of paper can be immersed, less is expected to be wasted. Additionally, to automate the process, a stamping machine is put into use instead. This is done using the ecoinvent process deep drawing, steel, 650kN press, single stroke as a proxy.

The changes in flows are shown in Table S13.

Table S13 *Differences in magnitudes of economic flows between scenarios for the paper device.*

Flow name	Laboratory	Commercial	Unit
[A2.2] Immersion process for 1 paper device			
Economic inputs			
paraffin	2.56E-01	2.45E-01	kilogram
Economic outputs			
municipal solid waste	2.56E-02	1.23E-02	kilogram
[A2.5] Manufacturing paper microfluidic device			
Economic inputs			
electricity, medium voltage	1.30E-03	0.00	unit
stamp	2.00E-04	0.00	kilowatt hour
deep drawing, steel, 650 kN press, single stroke	0.00	1.96E-03	kilowatt hour

PLA

In the case of the PLA device, it is unreasonable to 3D print many devices simultaneously, as the printing time takes so long. It can be argued that the advantages that are associated with 3D printing are the main reason to choose 3D printing, and not any other method. These advantages include low cost and rapid prototyping. However, in this scenario the production is modelled for the commercial scale, where these initial advantages are no longer relevant. While other commercialisation strategies that retain these advantages might exist, they are out of the scope of this study.

Therefore, in this model, the mass production of the PLA device is modelled using a different manufacturing method. Injection moulding still allows the use of the same material, and the same level of detail can be achieved as in the original design. Modelling large-scale production of the PLA device is therefore modelled by replacing the 3D printing processes for injection moulding, which is included in the ecoinvent database as a service per kilogram of material. It is assumed that the same amount of material is needed per device in this scenario. The changes in flows are shown in Table S14.

Table S14 *Differences in magnitudes of economic flows between scenarios for the PLA device.*

Flow name	Laboratory	Commercial	Unit
[A3.6] 3D printing PLA microfluidic device			
Economic inputs			

electricity, medium voltage, from scenario	6.32E+00	0.00	kilowatt hour
PLA filament	1.40E-02	0.00	kilogram
polylactide, granulate	0.00	1.40E-02	kilogram
injection moulding	0.00	1.40E-02	unit

Results

S4 Characterisation results

S4.1 Characterisation results per scale

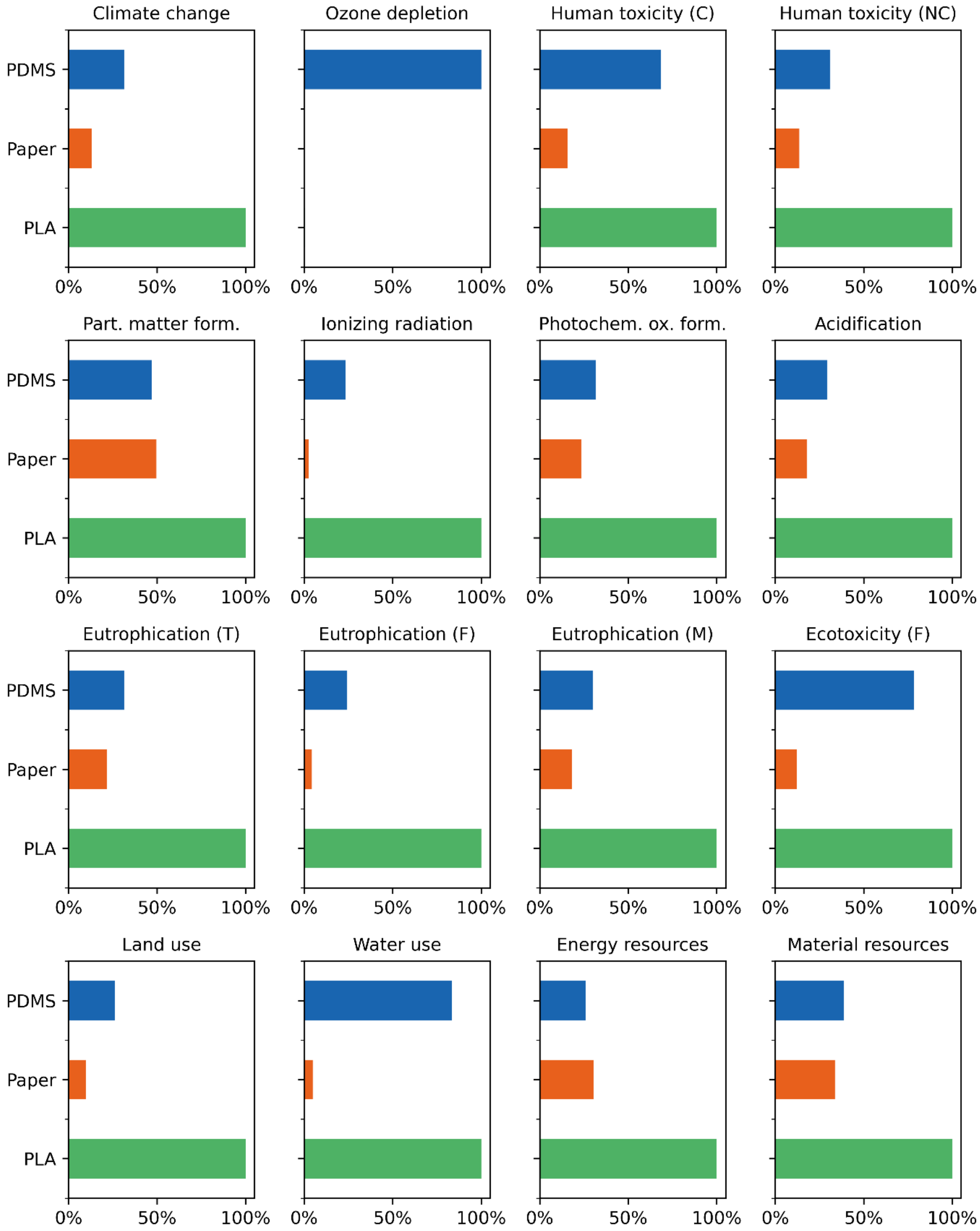

Fig. S9 *Characterisation results for the PDMS, paper, and PLA product systems, assuming a laboratory-scale production scenario.*

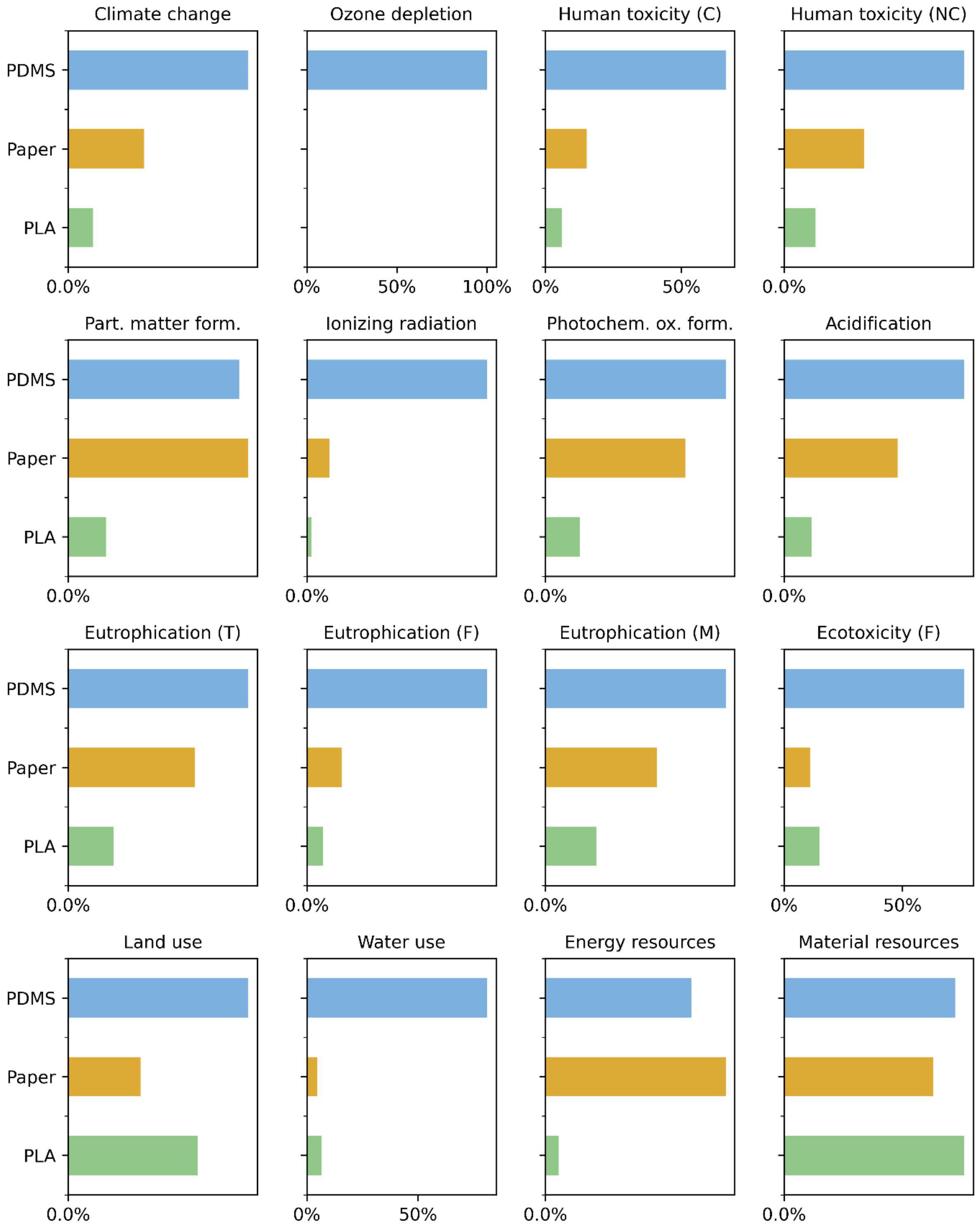

Fig. S10 *Characterisation results for the PDMS, paper, and PLA product systems, assuming a commercial-scale production scenario.*

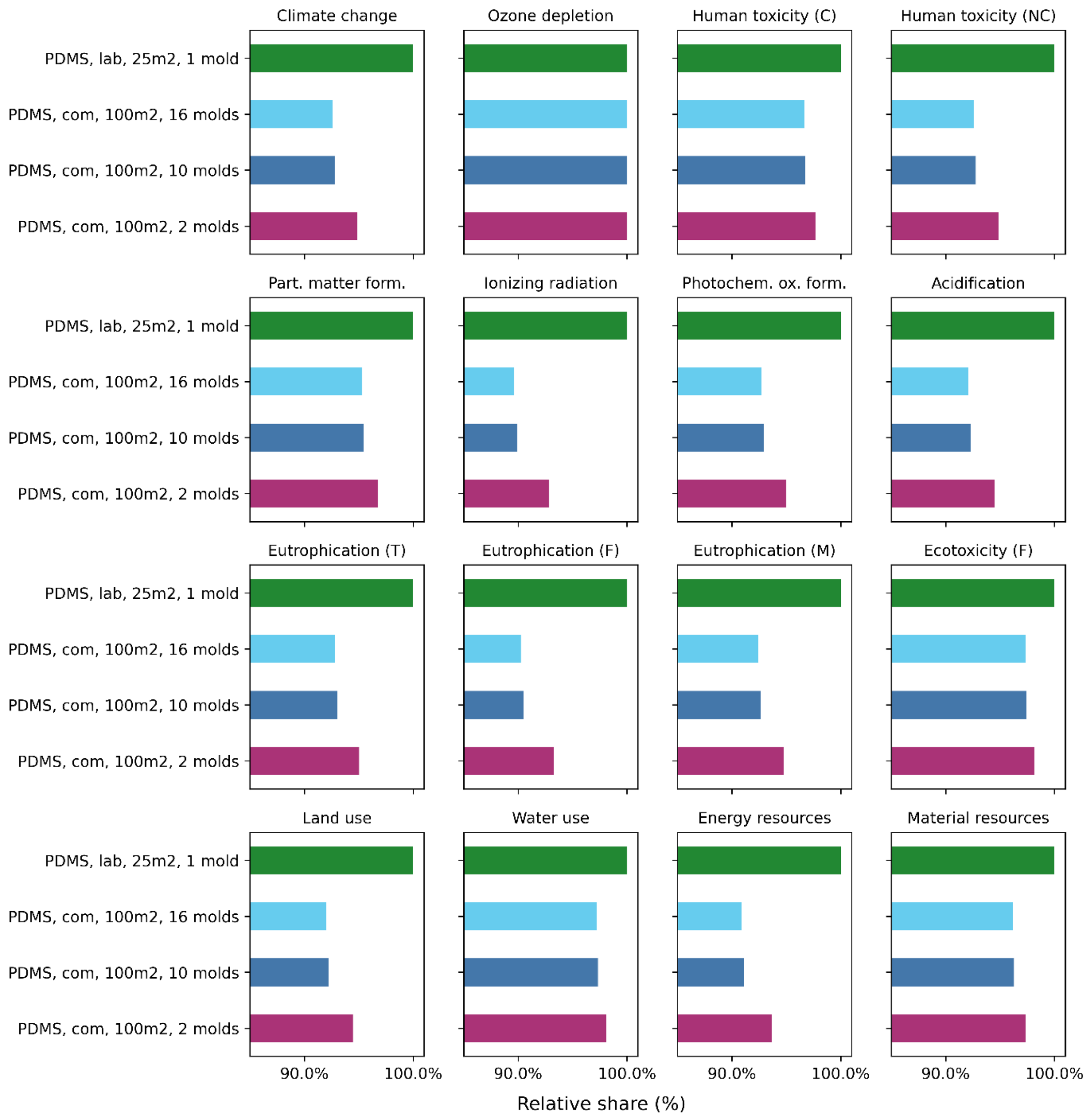

Figure S11 *Sensitivity analysis results for the PDMS device, assuming different production scenarios, cleanroom sizes, and number of molds produced.*

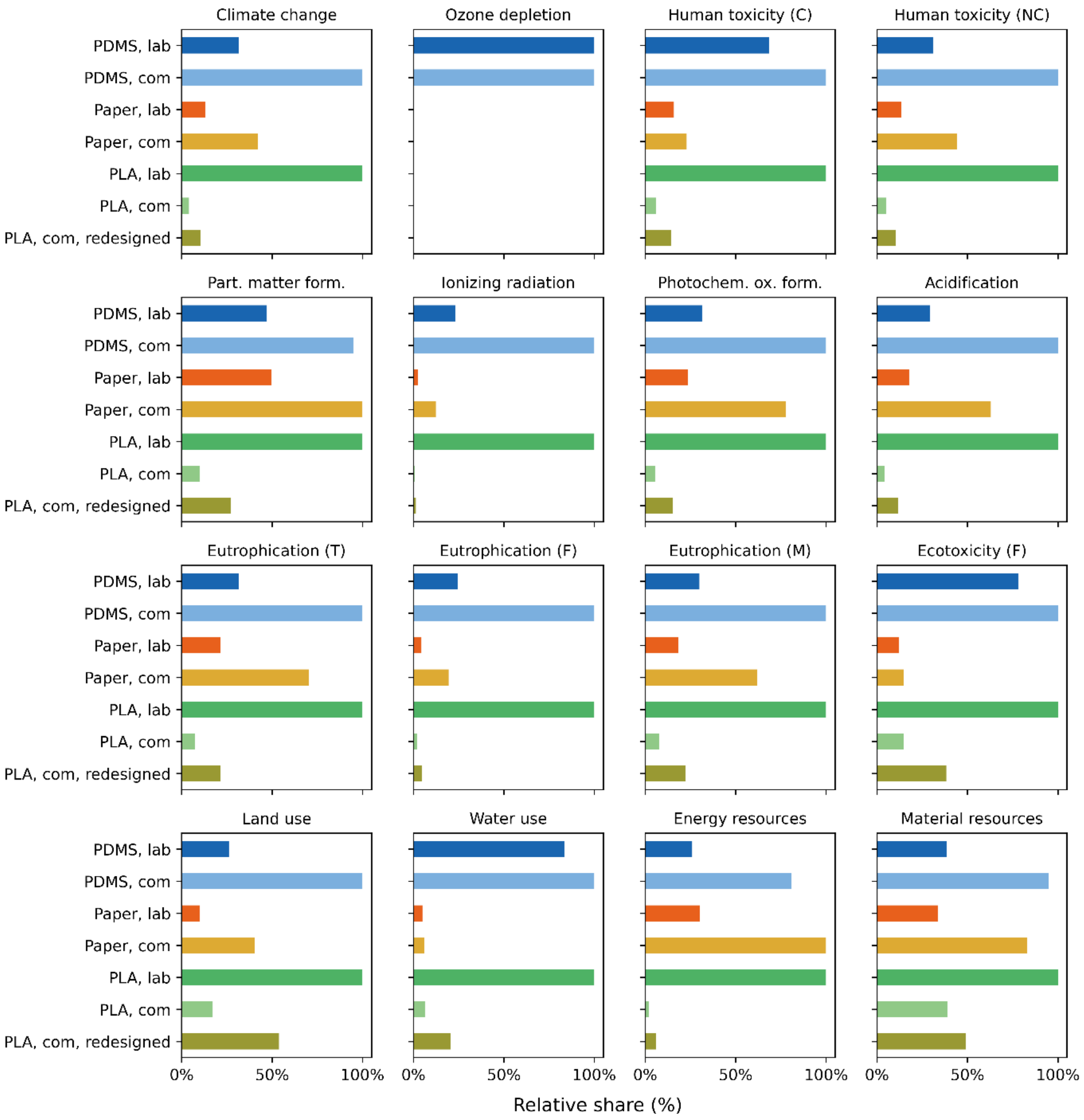

Figure S12 *Characterisation results for the PDMS, paper, and PLA product systems, for both laboratory and commercial scales. Sensitivity analysis was applied to assess the impact of potentially redesigning the PLA device in a way that would require thrice as much material.*

Table S15 *Characterisation results for the three alternatives: PDMS, paper, and PLA.*

Impact category	PDMS	Paper	PLA	Unit
climate change	6.71E-01	2.78E-01	2.13E+00	kg CO_2-Eq
ozone depletion	7.47E-06	3.11E-09	3.74E-08	kg CFC-11-Eq
human toxicity: carcinogenic	3.03E-09	6.99E-10	4.41E-09	CTUh
human toxicity: non-carcinogenic	6.01E-09	2.63E-09	1.93E-08	CTUh
particulate matter formation	1.89E-08	1.99E-08	4.00E-08	disease incidence
ionising radiation: human health	3.16E-01	3.54E-02	1.36E+00	kBq U_{235}-Eq
photochemical oxidant formation: human health	1.73E-03	1.30E-03	5.49E-03	kg NMVOC-Eq
acidification	3.18E-03	1.92E-03	1.08E-02	mol H+-Eq
eutrophication: terrestrial	5.29E-03	3.60E-03	1.67E-02	mol N-Eq
eutrophication: freshwater	4.53E-04	8.20E-05	1.85E-03	mol P-Eq
eutrophication: marine	5.79E-04	3.52E-04	1.93E-03	kg N-Eq
ecotoxicity: freshwater	5.65E+00	8.86E-01	7.22E+00	CTUe
land use	2.49E+00	9.45E-01	9.51E+00	dimensionless
water use	1.10E+00	6.51E-02	1.32E+00	m3 world Eq deprived
energy resources: non-renewable	1.28E+01	1.50E+01	4.94E+01	MJ, net calorific value
material resources: metals/minerals	2.89E-06	2.52E-06	7.49E-06	kg Sb-Eq

Table S16 *Characterisation results for the PDMS, paper, and PLA device for the case that they are manufactured on a large scale, such as for commercialization.*

Impact category	PDMS	Paper	PLA	Unit
climate change	6.21E-01	2.61E-01	8.53E-02	kg CO_2-Eq
ozone depletion	7.47E-06	3.04E-09	2.02E-09	kg CFC-11-Eq
human toxicity: carcinogenic	2.93E-09	6.72E-10	2.65E-10	CTUh
human toxicity: non-carcinogenic	5.56E-09	2.47E-09	9.71E-10	CTUh
particulate matter formation	1.80E-08	1.90E-08	3.99E-09	disease incidence
ionising radiation: human health	2.83E-01	3.50E-02	7.00E-03	kBq U_{235}-Eq
photochemical oxidant formation: human health	1.61E-03	1.25E-03	3.08E-04	kg NMVOC-Eq

acidification	2.93E-03	1.84E-03	4.51E-04	mol H+-Eq
eutrophication: terrestrial	4.91E-03	3.46E-03	1.24E-03	mol N-Eq
eutrophication: freshwater	4.09E-04	7.96E-05	3.65E-05	mol P-Eq
eutrophication: marine	5.36E-04	3.31E-04	1.52E-04	kg N-Eq
ecotoxicity: freshwater	5.50E+00	8.03E-01	1.08E+00	CTUe
land use	2.29E+00	9.20E-01	1.64E+00	dimensionless
water use	1.07E+00	6.33E-02	8.76E-02	m3 world Eq deprived
energy resources: non-renewable	1.16E+01	1.44E+01	1.06E+00	MJ, net calorific value
material resources: metals/minerals	2.78E-06	2.43E-06	2.93E-06	kg Sb-Eq

S4.2 Contribution analysis

The numerical values for the contribution analysis discussed in the **Results and Discussion** are provided here. These results are split up between the most contributing unit processes and economic flows. Those for the PDMS device are shown in Table S16 and Table S17 respectively. For the paper device, the analysis results are shown in Table S18 and Table S19 respectively, while for the PLA device they are shown in Table S20 and Table S21 respectively.

Table S17 *Contribution analysis for the PDMS device on lab scale, based on the economic flows of the product system. Values shown in percentages.*

**Impact category**	**Polydimethyl-siloxane**	**Oxygen, liquid**	**electricity, medium voltage**	**silicon mould**	**Other**
climate change	17.60	59.60	10.20	11.00	1.60
ozone depletion	95.40	0.00	0.00	4.45	0.15
human toxicity: carcinogenic	62.30	28.00	3.65	4.55	1.50
human toxicity: non-carcinogenic	24.80	60.10	10.20	3.87	1.03
particulate matter formation: human health	49.80	37.40	5.13	3.67	4.00
ecotoxicity: freshwater	70.70	21.70	2.93	3.23	1.44
material resources: metals/minerals	41.60	33.00	16.50	4.18	4.72
water use	0.00	91.60	3.01	0.00	5.39

Table S18 *Contribution analysis for the PDMS device on commercial scale, based on the economic flows of the product system. Values shown in percentages.*

**Impact category**	**Polydimethyl-siloxane**	**Oxygen, liquid**	**electricity, medium voltage**	**silicon mould**	**Other**
climate change	19.00	64.40	2.66	10.80	3.14
ozone depletion	95.40	0.00	0.00	4.45	0.15
human toxicity: carcinogenic	64.30	29.00	0.00	4.26	2.44
human toxicity: non-carcinogenic	26.80	64.90	2.23	3.12	2.95
particulate matter formation: human health	52.30	41.20	0.00	3.30	3.20
ecotoxicity: freshwater	72.60	22.30	0.00	0.00	5.10
material resources: metals/minerals	43.20	34.40	16.60	0.00	5.80
water use	0.00	94.20	0.00	0.00	5.80

Table S19 *Contribution analysis for the paper device on lab scale, based on the economic flows of the product system. Values shown in percentages.*

**Impact category**	**paraffin**	**electricity, medium voltage**	**municipal solid waste**	**filter paper**	**pipette**	**Other**
climate change	67.50	16.00	5.78	4.19	4.73	1.80
ozone depletion	43.20	24.60	0.00	12.00	14.70	5.50
human toxicity: carcinogenic	63.60	12.82	0.00	11.80	6.70	5.08
human toxicity: non-carcinogenic	67.80	15.14	6.31	0.00	5.82	4.93
particulate matter formation: human health	88.60	3.91	0.00	0.00	0.00	7.49
ecotoxicity: freshwater	55.20	15.00	15.40	6.30	5.76	2.34
material resources: metals/minerals	81.70	3.90	0.00	6.52	5.79	2.09

water use	42.30	41.10	0.00	6.87	5.79	3.94

Table S20 *Contribution analysis for the paper device on commercial scale, based on the economic flows of the product system. Values shown in percentages.*

Impact category	paraffin	electricity, medium voltage	municipal solid waste	filter paper	pipette	Other
climate change	68.60	16.80	2.94	4.45	5.02	2.19
ozone depletion	42.20	24.90	0.00	12.20	15.00	5.70
human toxicity: carcinogenic	63.30	13.20	0.00	12.30	6.98	4.22
human toxicity: non-carcinogenic	69.10	16.00	3.22	0.00	6.20	5.48
particulate matter formation: human health	88.80	4.10	0.00	0.00	0.00	7.10
ecotoxicity: freshwater	58.20	16.60	8.13	6.96	6.36	3.75
material resources: metals/minerals	81.00	4.01	0.00	6.77	6.01	2.21
water use	41.60	42.40	0.00	7.06	5.95	2.99

Table S21 *Contribution analysis for the PLA device on lab scale, based on the economic flows of the product system. Values shown in percentages.*

Impact category	electricity, medium voltage	disk drive	pipette	PLA filament	Other
climate change	96.40	0.00	0.00	0.00	3.60
ozone depletion	94.70	0.00	0.00	0.00	5.30
human toxicity: carcinogenic	94.30	0.00	0.00	3.51	2.19
human toxicity: non-carcinogenic	95.30	0.00	0.00	0.00	4.70
particulate matter formation: human health	90.90	0.00	0.00	6.93	2.17
ecotoxicity: freshwater	86.10	0.00	0.00	9.47	4.43
material resources: metals/minerals	60.90	34.10	0.00	4.30	0.70
water use	94.60	0.00	0.00	5.02	0.38

Table S22 *Contribution analysis for the PLA device on commercial scale, based on the economic flows of the product system. Values shown in percentages.*

**Impact category**	**polylactic acid, granulate**	**injection moulding**	**municipal solid waste**	**disk drive**	**pipette**	**glucose assay**	**Other**
climate change	52.70	21.40	11.10	9.51	5.13	0.00	0.16
ozone depletion	31.50	17.80	0.00	16.90	7.53	25.70	0.57
human toxicity: carcinogenic	48.00	14.80	3.80	27.10	5.89	0.00	0.41
human toxicity: non-carcinogenic	37.60	11.50	10.10	35.50	5.25	0.00	0.05
particulate matter formation: human health	59.50	18.60	6.31	11.90	3.54	0.00	0.15
ecotoxicity: freshwater	65.40	5.22	7.44	20.20	0.00	0.00	1.74
material resources: metals/minerals	0.00	0.00	0.00	87.00	0.00	0.00	13.00
water use	86.80	7.86	0.00	3.09	0.00	0.00	2.25

REFERENCES

1. Langhorst, T., Winter, B., Roskosch, D. & Bardow, A. Stoichiometry-Based Estimation of Climate Impacts of Emerging Chemical Processes: Method Benchmarking and Recommendations. *ACS Sustain. Chem. Eng.* **11**, 6600–6609 (2023).

2. Koh, A. *et al.* A soft, wearable microfluidic device for the capture, storage, and colorimetric sensing of sweat. *Sci. Transl. Med.* **8**, 366ra165 (2016).

3. Gabriel, E. F. M. *et al.* Highly sensitive colorimetric detection of glucose and uric acid in biological fluids using chitosan-modified paper microfluidic devices. *Analyst* **141**, 4749–4756 (2016).

4. Tothill, A. M. Developing a proof of principle 3D-printed lab-on-a-disc assay platform. (Cranfield University, 2017).

5. Riofrio, A., Alcivar, T. & Baykara, H. Environmental and Economic Viability of Chitosan Production in Guayas-Ecuador: A Robust Investment and Life Cycle Analysis. *ACS Omega* **6**, 23038–23051 (2021).

6. Rogalski, J., Fiedurek, J., Szczordrak, J., Kapusta, K. & Leonowicz, A. Optimization of glucose oxidase synthesis in submerged cultures of Aspergillus niger G-13 mutant. *Enzyme Microb. Technol.* **10**, 508–511 (1988).

7. Huber, E., Bach, V., Holzapfel, P., Blizniukova, D. & Finkbeiner, M. An Approach to Determine Missing Life Cycle Inventory Data for Chemicals (RREM). *Sustainability* **14**, (2022).

8. Geisler, G., Hofstetter, T. B. & Hungerbühler, K. Production of fine and speciality chemicals: procedure for the estimation of LCIs. *Int. J. Life Cycle Assess.* **9**, 101–113 (2004).

9. Hischier, R., Hellweg, S., Capello, C. & Primas, A. Establishing life cycle inventories of chemicals based on differing data availability (9 pp). *Int. J. Life Cycle Assess.* **10**, 59–67 (2005).

10. Kim, S. & Overcash, M. Energy in chemical manufacturing processes: gate-to-gate information for life cycle assessment. *J. Chem. Technol. Biotechnol. Int. Res. Process Environ. Clean Technol.* **78**, 995–1005 (2003).

11. Parvatker, A. G. & Eckelman, M. J. Comparative evaluation of chemical life cycle inventory generation methods and implications for life cycle assessment results. *ACS Sustain. Chem. Eng.* **7**, 350–367 (2018).

12. Roh, K. *et al.* Early-stage evaluation of emerging CO 2 utilization technologies at low technology readiness levels. *Green Chem.* **22**, 3842–3859 (2020).

13. Gong, J., Darling, S. B. & You, F. Perovskite photovoltaics: life-cycle assessment of energy and environmental impacts. *Energy Environ. Sci.* **8**, 1953–1968 (2015).

14. Seeger, M., Otto, W., Flick, W., Bickelhaupt, F. & Akkerman, O. S. Magnesium Compounds. in *Ullmann's Encyclopedia of Industrial Chemistry* 41–77 (John Wiley & Sons, Ltd, 2011). doi:10.1002/14356007.a15_595.pub2.

15. Yao, J., Zhu, B. & Yao, J. CN104478704A - Preparation method of anhydrous trisodium citrate - Google Patents. (2014).

16. Lian, J. Z. *et al.* Quantifying the present and future environmental sustainability of cleanrooms. *Cell Rep. Sustain.* **1**, 100219 (2024).

17. Torosyan, S. A. *et al.* Synthesis of fullerene-containing methacrylates. *Mendeleev Commun.* **22**, 199–200 (2012).

18. Otto, W. US3220960A - Cross-linked hydrophilic polymers and articles made therefrom - Google Patents. (1960).

19. Shafiee, S. A., Aarons, J. & Hamzah, H. A. Review—Electroreduction of Peroxodisulfate: A Review of a Complicated Reaction. *J. Electrochem. Soc.* **165**, H785–H798 (2018).

20. Greenwood, N. & Earnshaw, A. *Chemistry of the Elements*. (Butterworth-Heinemann, 1997).

21. Ettel, V. A. EP0052444A1 - A process of producing cobalt (II) chloride - Google Patents. (1980).

22. Roose, P. Methylamines. *Ullmanns Encycl. Ind. Chem.* 1–10 (2015) doi:10.1002/14356007.a16{_535.pub4.

23. Walker, J. J. & Johnston, J. M. C.—Tetramethylammonium hydroxide. *J. Chem. Soc.* **87**, 955–961 (1905).

24. de Tarso Garcia, P., Garcia Cardoso, T. M., Garcia, C. D., Carrilho, E. & Tomazelli Coltro, W. K. A handheld stamping process to fabricate microfluidic paper-based analytical devices with chemically modified surface for clinical assays. *RSC Adv* **4**, 37637–37644 (2014).

25. Havelange, S. *et al.* Phosphoric Acid and Phosphates. *Ullmanns Encycl. Ind. Chem.* 1–55 (2022) doi:10.1002/14356007.a19{_465.pub4.

26. DeAngelis, K. M. Phosphate buffer. (2007).

27. Cerdas, F., Juraschek, M., Thiede, S. & Herrmann, C. Life Cycle Assessment of 3D Printed Products in a Distributed Manufacturing System. *J. Ind. Ecol.* **21**, S80–S93 (2017).

28. Crahay, O. Towards the design of a paper-based microfluidic sensor for water quality monitoring and comparative life cycle assessment. (Ecole polytechnique de Louvain, Université catholique de Louvain, 2020).

29. Shakeri, A., Khan, S. & Didar, T. F. Conventional and emerging strategies for the fabrication and functionalization of PDMS-based microfluidic devices. *Lab. Chip* **21**, 3053–3075 (2021).

30. Akyazi, T., Basabe-Desmonts, L. & Benito-Lopez, F. Review on microfluidic paper-based analytical devices towards commercialisation. *Anal. Chim. Acta* **1001**, 1–17 (2018).
